# Single-cell transcriptomics of the ventral posterolateral nucleus-enriched thalamic regions from HSV-1-infected mice reveal a novel microglia/microglia-like transcriptional response

**DOI:** 10.1186/s12974-022-02437-7

**Published:** 2022-04-06

**Authors:** Olus Uyar, Juan Manuel Dominguez, Maude Bordeleau, Lina Lapeyre, Fernando González Ibáñez, Luc Vallières, Marie-Eve Tremblay, Jacques Corbeil, Guy Boivin

**Affiliations:** 1 Research Center in Infectious Diseases, CHU de Québec-Laval University Research Center and Department of Pediatrics and Microbiology, Faculty of Medicine, Laval University, Quebec City, QC Canada; 2 Research Center in Infectious Diseases, CHU de Québec-Laval University Research Center and Department of Molecular Medicine and Big Data Research Centre, Faculty of Medicine, Laval University, Quebec City, QC Canada; 3 Integrated Program in Neuroscience, McGill University, Montreal, QC Canada; 4 Neurosciences Unit, CHU de Québec-Laval University Research Center, Quebec City, QC Canada; 5 Department of Molecular Medicine, Laval University, Quebec City, QC Canada; 6 Division of Medical Sciences, University of Victoria, Victoria, BC Canada; 7 Department of Neurology and Neurosurgery, McGill University, Montreal, QC Canada; 8 Department of Biochemistry and Molecular Biology, The University of British Columbia, Vancouver, BC Canada; 9 Centre for Advanced Materials and Related Technology (CAMTEC), University of Victoria, Victoria, BC Canada

**Keywords:** Herpes simplex virus 1, Encephalitis, Transcriptomics, Antiviral immune response, Microglia

## Abstract

**Background:**

Microglia participate in the immune response upon central nervous system (CNS) infections. However, the role of these cells during herpes simplex encephalitis (HSE) has not been fully characterized. We sought to identify different microglia/microglia-like cells and describe the potential mechanisms and signaling pathways involved during HSE.

**Methods:**

The transcriptional response of CD11b^+^ immune cells, including microglia/microglia-like cells, was investigated using single-cell RNA sequencing (scRNA-seq) on cells isolated from the ventral posterolateral nucleus (VPL)-enriched thalamic regions of C57BL/6 N mice intranasally infected with herpes simplex virus-1 (HSV-1) (6 × 10^5^ PFUs/20 µl). We further performed scanning electronic microscopy (SEM) analysis in VPL regions on day 6 post-infection (p.i.) to provide insight into microglial functions.

**Results:**

We describe a novel microglia-like transcriptional response associated with a rare cell population (7% of all analyzed cells), named “in transition” microglia/microglia-like cells in HSE. This new microglia-like transcriptional signature, found in the highly infected thalamic regions, was enriched in specific genes (*Retnlg*, *Cxcr2*, *Il1f9*) usually associated with neutrophils. Pathway analysis of this cell-type transcriptome showed increased NLRP3-inflammasome-mediated interleukin IL-1β production, promoting a pro-inflammatory response. These cells' increased expression of viral transcripts suggests that the distinct “in transition” transcriptome corresponds to the intrinsic antiviral immune signaling of HSV-1-infected microglia/microglia-like cells in the thalamus. In accordance with this phenotype, we observed several TMEM119^+^/IBA-I^+^ microglia/microglia-like cells immunostained for HSV-1 in highly infected regions.

**Conclusions:**

A new microglia/microglia-like state may potentially shed light on how microglia could react to HSV-1 infection. Our observations suggest that infected microglia/microglia-like cells contribute to an exacerbated CNS inflammation. Further characterization of this transitory state of the microglia/microglia-like cell transcriptome may allow the development of novel immunomodulatory approaches to improve HSE outcomes by regulating the microglial immune response.

**Supplementary Information:**

The online version contains supplementary material available at 10.1186/s12974-022-02437-7.

## Background

Herpes simplex virus 1 (HSV-1), a member of the alphaherpesvirinae subfamily, establishes lifelong latent infections in trigeminal ganglia [1, 2]. Occasional reactivations of the virus usually result in cold sores [3]. However, this neurotropic virus sometimes causes life-threatening pathologies, such as herpes simplex encephalitis (HSE). HSE is the most frequent lethal sporadic acute form of viral encephalitis globally, with a mortality rate of 30% despite antiviral therapy. Of the surviving patients, half suffers significant neurological sequelae due to neuronal and glial damage [4, 5]. The present paradigm is that viral replication and an over reactive immune response are the two main factors contributing to CNS damages [6]. Additional studies are required to characterize in-depth the molecular pathways mediating the immune response during HSE. Moreover, understanding the molecular mechanisms that generate this inflammation would drive the development of efficacious immunomodulatory strategies.

One of the drivers of neuroinflammation is microglia, which usually protects the CNS against foreign agents [7–9]. Microglia are a heterogeneous population that consists of diverse subtypes with different properties depending on their localization in the CNS [10]. They are the first immune cells to encounter HSV-1 in the CNS. Upon recognizing a pathogen, microglial cells can change from a ramified to an amoeboid morphology and migrate toward infected cells. Increased cytokines/chemokines production by these cells restricts viral spread [11–13]. Conflicting results have been reported regarding the impact of microglia in different neuroinflammatory disorders, including HSE. TLR2-mediated activation of microglia inducing oxidative stress was shown to be responsible for neurotoxicity during HSV-1 infection. In contrast, an absence of microglia in the early stage of infection worsens the outcome of the disease, suggesting their beneficial role in orchestrating an early protective immune response by regulating type-I Interferon (IFN) response and peripheral immune cell infiltration [14–18]. Despite various studies characterizing the role of microglia in HSE, a transcriptomic study on these immune cells isolated from HSV-1-infected CNS is still missing.

Following the early microglial response to HSV-1, an important number of peripheral immune cells infiltrate the CNS. One of the most abundant peripheral immune cells found in the HSV-1-infected brain is monocytes. Despite their distinct ontogeny from the bone marrow (instead of embryonic yolk sac), monocytes share common characteristics with resident microglia [9, 19]. During CNS inflammation, infiltrating myeloid cells, mostly monocytes, can colonize the brain and transform into long-lived ramified microglia-like cells, previously termed bone-marrow-derived microglia, which are highly enriched in microglial markers [20, 21]. Because of their similar antigenic profiles, microglia and infiltrating monocytes are difficult to distinguish [22]. Several studies have shown that monocyte-derived macrophages contribute to HSE by phagocytosing-infected cells and releasing cytokines/chemokines, reactive oxygen species (ROS), and nitric oxide (NO) [23, 24]. However, an uncontrolled monocyte mediated-inflammatory response is also considered a potential threat, similar to an exaggerated microglial response, leading to CNS damage [25]. Here, we identified signature markers for monocytes and microglia, which allowed us to decipher their respective transcriptional responses upon HSV-1 infection.

Single-cell RNA sequencing (scRNA-seq) has become a frequently used tool in infectious disease research, allowing the identification of biomarkers and pathways to elucidate molecular mechanisms of immune regulation [26, 27]. Furthermore, the analysis of pathogen mRNA using scRNA-seq can permit the identification of their targets [28]. We combined scRNA-seq and electron microscopy to analyze the cellular transcriptomic signatures and visualize the phenotypic changes of microglia vs. bone-marrow-derived microglia-like cells during HSE.

We show that moderately infected thalamic areas harbor highly phagocytic reactive microglia. In contrast, highly infected thalamic regions accommodate microglia/microglia-like cells with impaired physiological functions, such as decreased phagocytic activity. Our scRNA-seq analysis further reveals a novel transcriptional signature that we defined as “in transition” microglia/microglia-like cells, in highly infected thalamus. Finally, we demonstrate that the “in transition” transcriptome corresponds to the immune response of HSV-1-infected microglia/microglia-like cells acquiring a pro-inflammatory phenotype. Our study provides novel insights into microglia/microglia-like cell response during HSE and reveals potential pathways implicated in the antiviral mechanisms of HSV-1-infected microglia.

## Methods

### Animals

Six-week-old male C57BL/6 N mice were purchased from Charles River, Canada. Mice were slightly anesthetized and infected with clinical HSV-1 strain H25 or recombinant HSV-1 (rHSV-1) in 20 µl of the minimum essential medium by intranasal inoculation [29]. Similar amounts of viral suspension were equitably administered to the right and the left mouse nostrils. Animals were acclimated to standard laboratory conditions for 1 week and housed three to four per cage.

### Evaluation of clinical signs of HSE

Mice were monitored for HSE-related signs: ruffled fur, ocular swelling, shaking movements, swollen forehead, breathing difficulties, bodyweight loss, and mortality twice daily for 14 days. Animals were sacrificed when a bodyweight loss equal to or greater than 20% was achieved, or a combination of two other obvious sickness signs were observed.

In our HSE mouse model, symptoms are correlated with the brain viral titers. Based on the evaluation of clinical signs, mice could be divided into two categories: moderately (weight loss, ruffled fur) and highly (weight loss, ruffled fur, swollen forehead ± neurological signs) infected mouse. Thalamus collected from these moderately or highly infected mice were called moderately or highly infected thalamus, respectively.

### Infectious viral titer measurements

Mice were anesthetized by intraperitoneal injection of a mixture of ketamine/xylazine (at doses of 90 mg/ml and 10 mg/ml, respectively) and sacrificed by intracardiac perfusion with cold 0.9% saline. Brains were harvested on day 6 p.i. and separated into four regions: olfactory bulb, cerebral cortex, hippocampus/hypothalamus, and hindbrain (cerebellum, pons, and medulla) to identify infected areas. The brain tissues were first homogenized, then viral titers for each of four HSV-1-infected-brain parts were determined by counting plaque-forming units (PFUs)/mg in Vero cells, as described elsewhere [30]. In another mouse experiment, following the dissection of the thalamus/hypothalamus region used for single-cell preparation, the rest of the brain tissues was homogenized, and viral titers were determined, as described previously.

### Immunostaining studies

Mice were sacrificed by intracardiac perfusion with cold 0.9% saline followed by a 4% paraformaldehyde (PFA) solution in 0.1 M borax buffer, pH 9.5, at 4 °C. Extracted brains were post-fixed for 24 h and then placed in 20% sucrose diluted in 4% PFA for 24 h. Fixed brains were cut in 25-μm coronal and sagittal sections on dry ice using a microtome (Reichert-Jung, Cambridge Instruments Company). Free-floating sections were prepared for immunostaining with different antibodies (Table 1, immunofluorescence), as described elsewhere [19]. Sections were mounted onto SuperFrost slides (Fisher Scientific) and coverslipped with Fluoromount-G (Electron Microscopy Sciences) for immunofluorescence (IF) studies. Confocal fluorescence microscopy images were captured using a Confocal Quorum WaveFX spinning-disk confocal microscope (Quorum Technologies) equipped with a Hamamatsu ImageEM camera (Hamamatsu Corporation). Images were acquired using Volocity 4 software (Perkin Elmer).

**Table 1:** Antibodies used for flow cytometry and immunofluorescence analyses

	Target	Host	Clone	Fluorochrome	Manufacturer
Flow cytometry	CD45	Rat	30-F11	APC-Cy7	BD Biosciences
	CD11b	Rat	M1/70	BUV737	BD Biosciences
	CD3ε	Hamster	145-2C11	PE-CF594	BD Biosciences
	B220	Rat	RA3-6B2	PerCP-Cy5.5	BD Biosciences
	Ly6C	Rat	AL-21	BV605	BD Biosciences
	Ly6G	Rat	1A8	FITC	BD Biosciences
	CD115 (Csfr1)	Rat	AFS98	APC	eBioscience
	Viability Stain 510	No	No	BV510	BD Biosciences
	CD16/CD32	Mouse	93	Unconjugated	BD Biosciences
Immunofluorescence	TMEM-119	Rabbit	28-03	Unconjugated	Abcam
	HSV-1/-2	Goat	Polyclonal	No	Bio-Rad
	HSV-1/-2	Mouse	Polyclonal	No	Bio-Rad
	CD68	Rat	FA-11	No	Bio-Rad
	CD3ε	Hamster	145-2C11	No	Invitrogen
	IBA-I (Aif1)	Mouse	20A12.1	No	Sigma-Aldrich
	NLRP3	Rat	768,319	No	Invitrogen
	IL-1β	Rabbit	Polyclonal	No	Novus Biologicals
	Goat IgG	Donkey	Polyclonal	Alexa647	JacksonImmunoResearch
	Goat IgG	Donkey	Polyclonal	Alexa546	Invitrogen
	Rat IgG	Goat	Polyclonal	Alexa546	Invitrogen
	Rat IgG	Goat	Polyclonal	Alexa488	Invitrogen
	Mouse IgG	Goat	Polyclonal	Alexa488	Invitrogen
	Mouse IgG	Donkey	Polyclonal	Alexa546	Invitrogen
	Rabbit IgG	Goat	Polyclonal	Alexa488	Invitrogen
	Rabbit IgG	Goat	Polyclonal	Alexa546	Invitrogen
	Hamster IgG	Goat	Polyclonal	FITC	JacksonImmunoResearch

### Tissue collection for electron microscopy

On days 0 and 6 p.i., a subset of animals (*n* = 3 mice per timepoint) was anesthetized with a mixture of ketamine hydrochloride and xylazine (at doses of 90 mg/ml and 10 mg/ml, respectively). Mice were blood-flushed by intracardiac perfusion with ~ 15 ml ice-cold phosphate-buffered saline (PBS, pH 7.4) and then perfused with ~ 75 ml ice-cold 3.5% (w/v) acrolein in 100 mM phosphate buffer (PB, pH 7.4), followed by ~ 150 ml ice-cold 4% (w/v) PFA in PB. Brains were extracted, post-fixed in ice-cold 4% (w/v) PFA in PB for 2 h and washed in 50 mM PBS-pH 7.4, three times for 10 min. Fixed brains were then sectioned into 50 µm-thick coronal sections using a vibratome (VT1200S, Leica) and stored in a cryoprotectant solution (30% (v/v) ethylene glycol, 30% (v/v) glycerol in PBS) at − 20 °C until use.

### Immunostaining for electron microscopy

Two sections containing the ventral posterior nucleus (VPN) of the thalamus (Bregma − 1.35 to − 1.46; stereotaxic atlas of Paxinos and Franklin 4th edition (Paxinos and Franklin, 2013)) were used for immunostaining against HSV-1. Sections were washed in 25 mM potassium-phosphate buffered saline (KPBS, pH 7.4) three times for 10 min. Sections were quenched with 0.3% (v/v) H_2_O_2_ in KPBS for 10 min and washed in KPBS three times for 10 min. Sections were incubated with 0.1% (w/v) NaBH_4_ in KPBS for 30 min and washed in KPBS three times for 10 min. Next, sections were incubated in a blocking solution consisting of 4% normal donkey serum (Sigma), 1% bovine serum albumin (Sigma), 0.03% Triton X-100 (Sigma) in KPBS at room temperature (RT) for 30 min. Sections were finally incubated with goat anti-HSV1/2 (Bio-Rad) in KPBS (1:1000) at 4 °C overnight. On the following day, tissues were washed in KPBS five times for 5 min before incubation with donkey anti-goat 1.4 nm Nanogold-conjugated (Nanoprobes, Yaphank, NY, USA) secondary antibody (1:50) in KPBS at 4 °C overnight. The next day, brain sections were washed in KPBS five times for 5 min then twice for 5 min in freshly prepared 3% (w/v) sodium acetate solution. Staining was then revealed for 1 min at RT by silver enhancement (Nanoprobes). Sections were quickly rinsed in 3% sodium acetate solution, further rinsed in PB three times for 5 min, and washed in PBS five times for 3 min.

### Tissue processing for electron microscopy

After staining, tissues were post-fixed with osmium tetroxide and thiocarbohydrazide for scanning electron microscopy imaging. Brain sections were first immersed in a mixture (1:1) of 3% potassium ferrocyanide (BioShop, Burlington, ON, Canada) in PB and 4% aqueous osmium tetroxide (Electron Microscopy Sciences) at RT for 1 h and rinsed in Milli-Q water five times for 3 min. Sections were placed in 1% thiocarbohydrazide solution (Electron Microscopy Sciences) at RT for 20 min and rinsed in Milli-Q water five times for 3 min. Sections were then placed in 2% aqueous osmium tetroxide at RT for 30 min and washed in Milli-Q water five times for 3 min. Sections were further dehydrated in ascending ethanol concentrations (twice in 35%, once in 50%, once in 70%, once in 80%, once in 90%, three times in 100%), followed by propylene oxide (three times) for 5 min each. Sections were finally embedded in 100% Durcupan ACM resin (Electron Microscopy Sciences) for 24 h, then mounted between labeled ACLAR sheets (Electron Microscopy Sciences) embedded in a thin layer of resin and let to polymerize at 55 °C for 3 days. After resin polymerization, one of the ACLAR sheets was removed. The ventral posterior nucleus (VPN) of the thalamus was excised and glued on top of a resin block for ultramicrotomy. Ultrathin (~ 70 nm) sections were cut with an ultramicrotome (Ultracut UC7, Leica Biosystems), collected to a silicon nitride chip (Electron Microscopy Sciences), and glued on specimen mounts (Electron Microscopy Sciences). Samples were examined by array tomography at 5 nm (x, y) resolution using a Crossbeam 540 (GeminiSEM, Zeiss) field emission scanning electron microscope, with an acceleration voltage of 1.4 kV and current of 1.2 nA.

### Microglial analysis at the nanoscale resolution

Microglia/microglia-like cells were identified by their irregular nuclei with a dark heterogeneous chromatin pattern and an irregular cytoplasm, as well as frequent long stretches of endoplasmic reticulum cisternae and lipidic inclusions (i.e., lipofuscin, lysosomes), among other distinctive ultrastructural features [31]. Microglial/microglial-like cell ultrastructural analysis was performed using ImageJ software by an experimenter blind to the experimental conditions (*n* = 36–46 microglial cell bodies/group, *N* = 3 animals/group). Microglial organelles–lysosomes, lipidic inclusions, endosomes, endoplasmic reticulum, and Golgi apparatus cisternae, and mitochondria—as well as microglial interactions with their microenvironment—neurons, astrocytes, oligodendrocytes, microglia-like cells, synaptic elements, dark cellular processes, myelinated axons, myelin, blood vessels, and extracellular space pockets—were first qualitatively and quantitatively analyzed [32]. Afterward, HSV-1 nanogold particles were quantitatively analyzed in the microglia/microglia-like cells; the number of nanogold particles was counted, and the specific sub-compartment (organelles) for each analyzed particle was identified. Microglia/microglia-like cells were considered immunostained when at least two nanogold particles were visible within their nucleus, cell body, or plasma membrane. In addition, their contacts with infected cells identified by immunostaining were also assessed.

Lysosomes were identified by their dense heterogeneous contents enclosed by a single well-defined membrane and classified into immature or mature lysosomes. The latter is fused with endosomes and possesses lipofuscin granules (oval structure of finely granular composition with a fingerprint-like pattern) [33–35]. Endosomes were categorized as empty and digested, while their total number was also determined. Dilation of the endoplasmic reticulum or Golgi apparatus was characterized by a distance between cisternae greater than 50 nm [36]. In addition to determining the total count of mitochondria, mitochondrial abnormalities that included elongated (length > 1 µm) and donut-shaped mitochondria [32, 37] were quantified.

Synapses were recognized by the contact between a presynaptic axon terminal containing synaptic vesicles and a postsynaptic spine, often with a visible postsynaptic density at their junction. Dark processes, which can belong to microglia/microglia-like cells, among other cell types, were also examined. These structures show a condensed cytoplasm and frequently contain organelles showing alterations associated with cellular stress. Myelinated axons were recognized by their compact and electron-dense myelin sheath surrounding the axons. Myelin abnormalities were also identified, including myelin decompaction (distancing of myelin sheaths) and degenerating myelin (ballooning, swelling, or degradation). Neurons were distinguished by pale nuclei and cytoplasm, often with apical dendrite and innervation from axon terminals. Like neurons, astrocytes are characterized by pale nuclei and cytoplasm but recognized by their nuclear heterochromatin pattern that forms a thin rim on the edge and their irregular and angular cytoplasm that often contains intermediate filaments. Similar to microglia/microglia-like cells, oligodendrocytes were identified by their dark nuclei with a heterogeneous chromatin pattern and a dark squarish or rectangular-shape cytoplasm, often containing short and wide endoplasmic reticulum cisternae organized in the vicinity of the nucleus and ribosomes, as well as broader space between nuclear membranes than microglia/microglia-like cells [31]. Finally, extracellular space pockets were quantified directly juxtaposed with microglia/microglia-like cells. Extracellular space pockets containing degraded elements or debris were designated as extracellular digestion, also known as digestive “exophagy” [31, 38, 39].

### Flow cytometry analyses of brain homogenates

Mice were deeply anesthetized as described above and perfused intracardially with ice-cold DPBS (Dulbecco’s phosphate-buffered saline, pH 7.4) without Ca^2+^ and Mg^2+^ before harvesting brains. The thalamic/hypothalamic region was dissected and chopped into fine bits using a razor blade. For the dissociation, minced tissue was incubated in DPBS without Ca^2+^ and Mg^2+^ containing papain (20 units/ml, prepared as described in the manufacturer’s instructions) and DNase-I (100 μL/ml) for 30 min at 37 °C. Homogenates were filtered through a 100 µm then 70 µm cell strainer (BD Biosciences). After a washing step, cells were centrifuged at 600×*g* for 40 min in a Percoll gradient (37% and 80%) (GE Healthcare). The cell ring at the interphase was collected, centrifuged at 300 × *g* for 10 min, and washed twice with DPBS containing 2% fetal bovine serum (FBS; Wisent). Cells were incubated with CD16/CD32 (Mouse BD Fc Block™ 2.4G2) and FVS.510 (viability) on ice for 30 min. After a washing step, cells were incubated with a pool of antibodies (Table 1, flow cytometry). For CD115 staining, cells were washed once, permeabilized with eBioscience™ Foxp3/Transcription Factor Staining kit, and incubated for 60 min, following the first incubation with the antibody pool. Labeled cells were fixed, washed, and resuspended in DPBS. Flow cytometry analyses and data acquisition were performed using a BD SORP LSR II and BD FACSDiva software, respectively.

### Single CD11b^+^ cell suspension for sequencing

For single-cell sequencing, mice were transcardially perfused with DPBS, and brains were quickly dissected on ice. As described above, the minced thalamic/hypothalamic regions were enzymatically digested with papain. The tissue was then gently mashed through a 70 µm cell strainer to have a single cell suspension. The cell suspension was washed with ice-cold DPBS without C^2+^ and Mg^2+^ containing 20 mM ethylenediaminetetraacetic acid (EDTA) and 5% of heat-inactivated FBS. As described previously, the cell ring from the Percoll gradient step was collected, centrifuged, and washed twice with magnetic-activated cell sorting (MACS) buffer. Ten μL of CD11b (Microglia) MicroBeads (Miltenyi) were added into 90 μL of cell suspension. Following 15 min of incubation at 4 °C, cell suspension with magnetic beads was applied onto MS MAC columns to isolate CD11b^+^ microglia/microglia-like cells and immune cells, as described in the manufacturer’s instructions. CD11b^+^ cells were counted manually using trypan blue with a standard hemocytometer to measure cell suspension concentration and cell viability. For each sample, 5 × 10^3^–1 × 10^4^ cells incubated with FVS.510 viability stain were also analyzed by BD SORP LSR II to confirm the % of viability. Cells were then centrifuged and resuspended in DPBS to have a final concentration of 700–1000 cells/µl, required for Chromium (10 × Genomics) single-cell sequencing.

### Single-cell library construction and sequencing

Single cells from two independent experiments of two (one infected and one uninfected) and three (two infected and one uninfected) samples each were processed using Chromium™ Next GEM Single Cell 3′ GEM, Library & Gel Bead Kit v3.1 Kit following the manufacturer’s user guide (10 × Genomics, Pleasanton, CA). After MACS sorting, cells were spun down for each experiment, resuspended in 100 µl of PBS, and subjected to cell quality control using BioRad TC10 automated cell counter (Bio-RAD, Mississauga, Canada). All processed samples had cell viability > 80%. After determining cell density, cells of each sample were injected into channels, aiming to achieve ~ 3000 cells per channel. Gel Beads-in-Emulsion (GEMs) were formed in channels of a chip in the 10 × Chromium instrument and then collected into an Eppendorf plate for GEM reverse transcription (GEM-RT) reaction. After GEM clean up, GEM-RT products were subjected to cDNA amplification for 13 cycles using poly(dT) primers followed by a 3’ gene expression library construction using 10X specific primers. (10 × Genomics, Pleasanton, CA) and by SPRIselect (Beckman Coulter, Brea, CA) beads clean up. The quality of libraries was examined with a DNA screen tape D5000 on a TapeStation 2200 (Agilent Technologies, Santa Clara, CA, USA), and the quantification was done on the QuBit 3.0 fluorometer (ThermoFisher Scientific, Canada). Subsequently, libraries with unique index were pooled together in equimolar ratio and sequenced for paired-end 100 pb sequencing on a NovaSeq 6000 (Novaseq 6000 S1 reagent kit (200 cycles), Illumina Inc., San Diego, CA, USA) at the Next-Generation Sequencing Platform, Genomics Center, CHU de Québec-Université Laval Research Center, Québec City, Canada. The coverage was approximately 100 million paired-end reads/sample.

### scRNAseq data and pathway analysis

Sequencing data were processed, aligned to the mm10 reference genome, and aggregated into a single file using Cell Ranger v6.0 (10 × Genomics) with default settings. This file was examined using Loupe Browser v5.0.1 (10 × Genomics) and filtered to remove low-quality cells (i.e., with < 1000 UMIs or > 15% of reads from mitochondrial genes) as well as ambiguous cells (i.e., doublets and phagocytosed cells identified based on co-localized cell-specific markers (e.g.,* Hbb-bt*, *C1qa*, *Ly6g*, *Cd3e*, *Cd19*, *Sdc1,* etc.). A re-analysis following the resulting selection was launched using the reanalyze option from the Cell Ranger software. Downstream analyses were performed on the filtered data reanalyzed with Cell Ranger using R version 4.0.5. The data were first processed by Seurat v.4.04 [40]. Data normalization was done with a scale factor of 10,000. The identification of the barcodes to the clusters was then imported by the Seurat object. Differential Expression Tests were done using MAST v.1.18. [41]. *P* values were corrected using the Benjamini–Hochberg method (BH) and filtered with a 5% false discovery rate (Q < 0.05). Cell subsets were identified using t-distributed stochastic neighbor embedding (tSNE) distribution and cell type-specific markers. Non-immune cells and contaminant blood cells (4% of whole scRNA-seq data) were identified and eliminated before further scRNAseq data analysis. Differentially expressed mRNAs were identified by significant feature comparison. A list of 6 neutrophil-related genes common to multiple diseases was used to show the neutrophil-like phenotype of “in transition” microglia/microglia-like cells [42]. UMAP dimensional reduction was used to visualize and verify clusters.

Pathway analysis was done using ReactomePA v.1.36. [43] The enrichPathway command was used while using the org.Mm.eg.db database. Only pathways that showed a *P* value of < 0.05 were considered, and the correction was done using BH. Visualization was obtained using the enrichplot package v. 1.12.2. and ggplot2 v.3.3.5. [44, 45].

### HSV-1 viral transcripts analysis

A new reference was created to analyze the viral transcripts by combining the mouse reference (mm10, July 2020) with the genome of herpesvirus 1 strain 17 (Genbank Accession: JN555585). We used *cellranger mkref* (10 × Genomics) to create the reference, then used Cellranger Count for each sample. The resulting outputs were then used for the *cellranger aggr* command to get an aggregated count matrix and then filtered by the *cellranger reanalyze* command using the chosen barcodes for this study. The resulting filtered count matrix was then exported to Seurat for analysis and visualization.

## Results

### Intranasally administered HSV-1 H25 strain is mostly found in the thalamus and hindbrain on day 6 post-infection

**Fig. 1:**
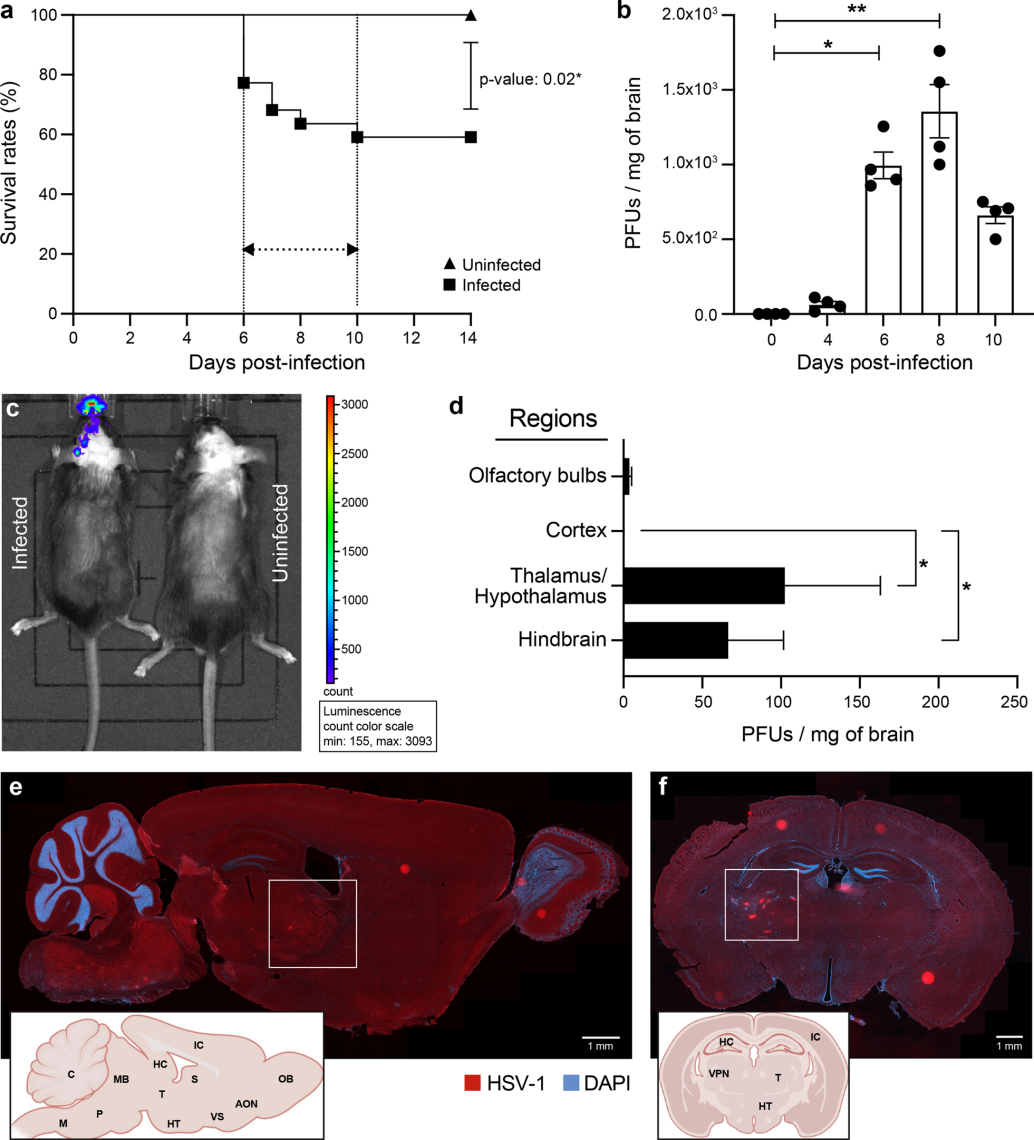
HSV-1 replication in thalamus and hindbrain leads to increased mortality on day 6 p.i. 6-week-old C57BL/6N male mice (*n* = 12 mice) were intranasally infected with 6 × 10^5^ PFUs of HSV-1 strain H25 in 20 μl MEM. **a** Survival curves of HSV-1-infected vs. uninfected control mice. Survival rates were analyzed using a log-rank (Mantel–Cox) test. **b** Viral titers in homogenates of brains were measured by a standard plaque assay on Vero cells on days 0, 4 and 6 p.i.. The results are reported as PFUs per milligram (mg) of brain homogenates and represent the means ± SEM for 4 mice per group at each timepoint. **c** Representative bioluminescence image of mouse infected with 6 × 10^5^ PFUs of recombinant HSV-1 (rHSV-1) on the left and uninfected control mouse on the right side, on day 6 p.i. The bioluminescent signal is expressed in average radiance (p/s/cm^2^/sr). **d** Identification of HSV-1^+^ brain regions on day 6 p.i. Viral titers in homogenates of different brain regions were measured by a standard plaque assay on Vero cells. Representative sagittal (**e**) and coronal (**f**) brain sections illustrating the localization of HSV-1 proteins (red) on day 6 p.i. Brain sections were immunostained with a primary polyclonal rabbit anti-HSV-1/2 antibody and a secondary Alexa-594 conjugated anti-rabbit antibody, followed by staining with DAPI (blue) (scale bar = 1 mm). HSV-1 signal was localized in the ventral posterolateral nucleus (VPL) of the thalamus (white squares) and hindbrain (cerebellum, medulla, and pons). Brain regions are indicated on the representative sagittal and coronal mouse brain sections inserted in the left corners (AON.: Anterior olfactory nucleus, C.: Cerebellum, HC.: Hippocampus, HT.: Hypothalamus, IC.: Isocortex, M: Medulla, MB.: Midbrain, OB.: Olfactory bulb, P.: Pons, S: Septum, T.: Thalamus, VPN: Ventral posterior nucleus, VS.: Ventral striatum). **f** All statistical analyses were performed using Kruskal–Wallis with “Dunn’s multiple comparisons test.” Statistically significant results are indicated as follows: *P < 0.05; **P < 0.01

We inoculated 6-week-old C57BL/6N mice intranasally with 6 × 10^5^ PFUs of HSV-1 H25 clinical strain. Infected mice developed a swollen forehead as their first clinical sign of the HSE on day 5 p.i. Mortality started to occur on day 6 p.i. (22.8%), then culminated on day 10 p.i. (41%). The overall mortality rate was significantly higher in the infected group (59%) than in uninfected mice (0%; *P* = 0.02) (Fig. 1a). On day 4 p.i., viral titers in whole brain homogenates became detectable (64 PFUs/mg of the brain). On day 6 (995 PFUs/mg of the brain), they significantly increased and reached the highest levels on day 8 p.i. (1357.5 PFUs/mg of the brain), then started to decrease on day 10 p.i. (662 PFUs/mg of the brain) (Fig. 1b).

To determine whether HSV-1 invades the CNS following intranasal infection, we imaged C57BL/6N mice on day 6 after intranasal infection with 6 × 10^5^ PFUs of rHSV-1 expressing Gaussia Luciferase using an intravital imaging system (IVIS) [29]. We first noted an ongoing HSV-1 infection in the nasal cavity. Widespread viral dissemination, mainly on the left side of the brain, was observed. The caudal forebrain, midbrain, and hindbrain were the three main regions emitting significant levels of bioluminescence (Fig. 1c). Interestingly, the hindbrain signal was more intense than the olfactory bulbs, considered the first region to get infected by HSV-1 in our HSE mouse model. This observation suggests that the thalamic infection occurred earlier than in the other areas, or the immune response in the olfactory bulbs was more effective at controlling the viral infection.

To provide further insights into the HSV-1 dissemination in the CNS, viral titers were next measured in four specific regions of the brain. We found that viral replication occurred mainly in the thalamic–hypothalamic regions (102.3 PFUs/mg of the brain) and hindbrain (66.3 PFUs/mg of the brain) on day 6 p.i. (Fig. 1d). Furthermore, we aimed to determine the exact location of HSV-1-infected CNS cells. To do so, we performed IF analyses on coronal and sagittal brain sections obtained from HSV-1-infected mice on day 6 p.i. In concordance with viral titers, the thalamus, hypothalamus, cerebellum, pons, and medulla were highly stained for HSV-1 (Fig. 1e). Coronal sections from the same group of infected mice confirmed that HSV-1^+^ CNS cells were mainly localized in the thalamus, specifically the VPL, a subregion of VPN (Fig. 1f). Our results suggest that distinct CNS regions display a differential vulnerability to HSV-1 infection. Based on these findings, we decided to study the highly infected thalamus, especially the VPL, on day 6 p.i.

### Reactive microglia are involved in the phagocytosis of HSV-1-infected cells and antigen presentation in moderately infected thalamic regions

**Fig. 2:**
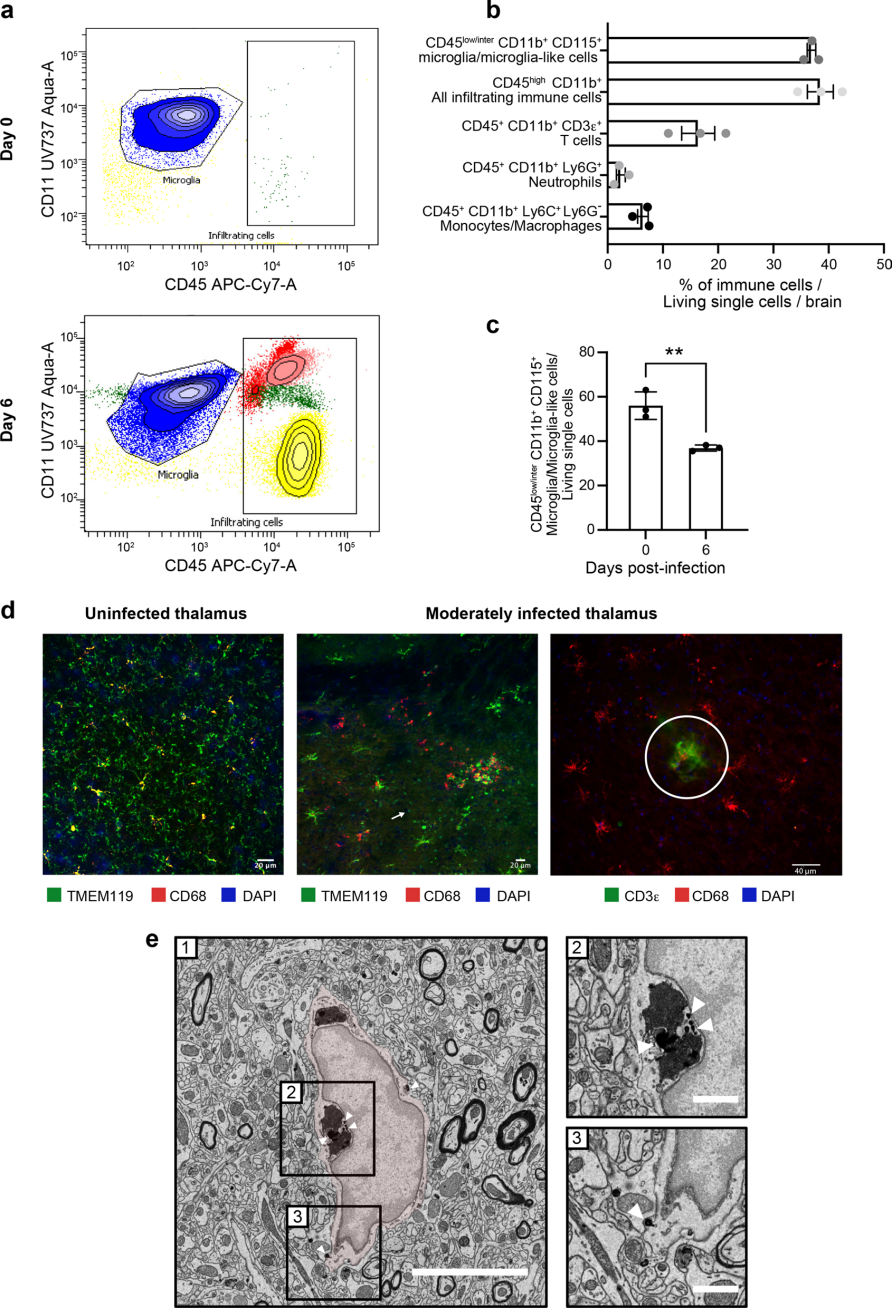
Reactive microglia are involved in the HSV-1^+^ cell clearance and antigen presentation in moderately infected thalamus. **a** Representative flow cytometry plots for CD45^+^CD11b^+^ gate showing infiltrating immune cell populations (T cells represented in yellow, neutrophils in red, monocytes/macrophages in light pink) and microglia/microglial-like cell (in blue) in whole brain homogenates on day 0 (top) and day 6 p.i. (bottom). **b** Percentages of different immune cell populations, compared to all living single cells obtained from a whole brain on day 6 p.i. **c** Mice infected with HSV-1 showed reduced percentages of CD45^+^CD11b^+^CD115^+^ microglia/microglial-like cells in the thalamus on day 6 p.i. The results represent the means ± SEM for 3 mice per group at each timepoint. Statistical analyses were performed using an unpaired *t* test. Statistically significant results are indicated as follows: **P < 0.001. **d**, left) Immunofluorescence image of uninfected VPL (day 0) immunostained for TMEM119^+^ (green) CD68^+^ (red) shows surveillant microglia. **d**, middle) Immunofluorescence analyses of brain sections of moderately infected VPLs on day 6 p.i. show TMEM119^+^ (green) CD68^+^ (red) microglia/microglial-like cells gathering around HSV-1^+^ cells (pink). TMEM119^+^ ameboid microglia/microglial-like cells (white arrow) are visualized. **d**, right) Fluorescent microscopy image shows colocalized signals of TMEM119^+^ (red) microglia/microglial-like cells establishing a direct contact with CD3e^+^ (green) T cells (white circle). Nuclear staining was done with DAPI for both images. **e** HSV-1^+^ nanoparticles are specifically found within the endosome and at the membrane of microglia/microglia-like cells in SEM images of VPL on day 6 p.i. On the representative picture of HSV1-immunopositive microglia/microglial-like cells (e-1), the microglia/microglial-like cell are pseudocolored in red, and square boxes identify subcellular compartments. (e-2, 3). Insert at higher magnification shows nanogold particles within the (e-2) endosomes containing digested contains and (e-3) at the cellular membrane. Scale bars are equivalent to 5 μm at low magnification (e-1) and 1 μm at higher magnification (e-2, 3)

To determine the CNS composition of immune cells on days 0 and 6 p.i., we analyzed HSV-1-infected whole brains by flow cytometry. As expected, the only immune cells observed before infection were CD45^int^CD11b^+^CD115^+^ microglia (Fig. 2a, top). On day 6 p.i., however, we observed three other types of leukocytes, i.e., CD45^+^CD11b^+^Ly6C^+^Ly6G^−^ monocytes/macrophages, CD45^+^CD11b^+^CD3ε^+^ T cells and CD45^+^CD11b^+^Ly6G^+^ neutrophils (Fig. 2a, bottom; Fig. 2b). Interestingly, the number of microglia-like cells was significantly reduced (Fig. 2c, *P* = 0.006), suggesting a possible elimination or phenotypic transformation of reactive microglia during HSE. We conclude that infiltrating immune cells and microglia/microglia-like cells are jointly involved in HSE control.

IF studies were subsequently performed in the infected VPN and its subregion VPL of the thalamus on day 6 p.i.. We focused on this thalamic region connecting the trigeminal nerve via the trigeminothalamic tract, which HSV-1 potentially uses to infiltrate the CNS [46]. The microglia/microglia-like cell populations were labeled using TMEM119, a well-known microglia marker [47]. Qualitative analysis of confocal images of moderately infected thalamus showed that TMEM119^+^ cells underwent morphological alterations, such as swollen cell bodies and thicker cytoplasmic processes, compared to surveillant microglia prior to the infection (Fig. 2d, left and middle). Besides these hypertrophic/reactive microglia, we also observed important numbers of TMEM119^+^ ameboid microglia/microglia-like cells, corresponding to a hyperreactive microglial phenotype (Fig. 2d, middle). Microglia/microglia-like cells migrated towards HSV-1^+^ cells and were closer to one another in the moderately infected thalamus on day 6 p.i. The increased clustering of microglia/microglia-like cells around infected cells was associated with a decreased microglial density in the surrounding parenchyma (Fig. 2d, middle and right). We also found TMEM119^+^ microglia/microglia-like cells expressing the phagolysosomal activity marker CD68 in moderately infected VPLs, suggesting microglial involvement in viral clearance by phagocytosis of HSV-1^+^ cells. Of note, most CD68^+^ spots were not colocalized within TMEM119^+^ cells, indicating the presence of other phagocytic cells with even higher lysosomal activity in the region (Fig. 2d, middle). Some of the hypertrophic/reactive microglia/microglia-like cells closely localized with CD3ε^+^ T cells (Fig. 2d, right), suggesting potential antigen uptake and processing for microglial antigen presentation.

**Fig. 3:**
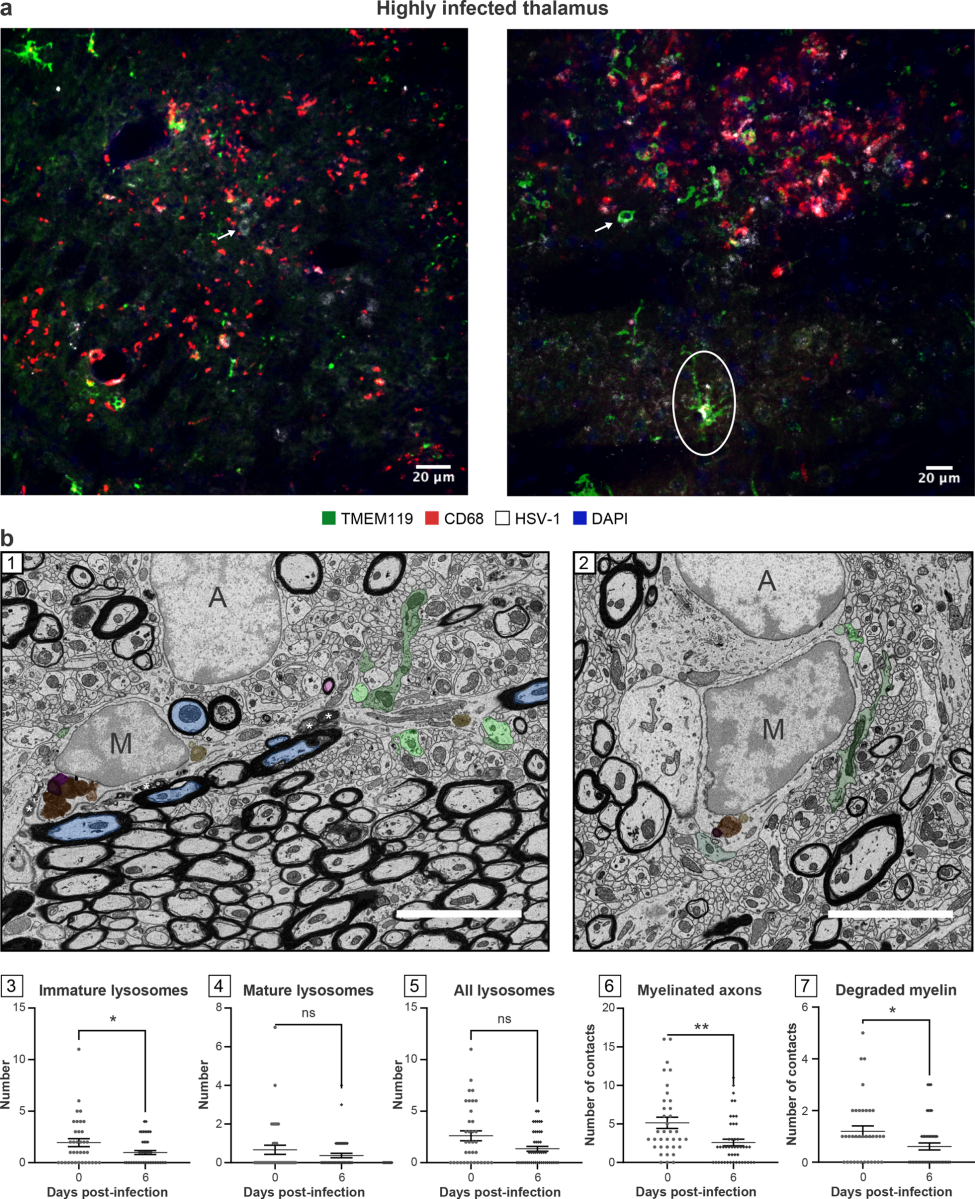
TMEM119^+^ microglia/microglial-like cells exhibit impaired functions, including reduced phagocytic activity in highly infected thalamus. **a** HSV-1-infected thalamus was labeled with antibodies against TMEM-119 (green), CD68 (red), HSV-1 (white), and counterstained with DAPI (blue). (left) Highly infected thalamus immunostained with TMEM119 (green), CD68 (red), HSV-1 (white) antibodies, and counterstained with DAPI showed increased numbers of TMEM119^−^/CD68^+^ cells in close/direct contact with HSV-1^+^ CNS cells (white arrow) on day 6 p.i.. (right) Confocal microscopy image shows TMEM119^+^ ameboid microglia/microglia-like cells (white arrow) with the lack of CD68 expression. HSV-1 signal was colocalized with TMEM119^+^/CD68^−^ ramified (white circle) and ameboid microglia/microglia-like cells (white arrow), suggesting an impaired phagocytic activity following microglial infection by HSV-1 or the phagocytosis of HSV-1^+^ CNS cells. **b** (1, 2) Representative pictures at day (1) 0 and (2) 6 p.i. showed that on day 6 p.i. microglia/microglial-like cells have a lower number of (3) immature lysosomes without major changes in (4) mature and (5) total lysosomes number and made fewer contacts with (6) myelinated axons and (7) degraded myelin. (1, 2) Microglia/microglial-like cell organelles and elements of their microenvironment that are in contact with the cell body are pseudocolored; immature lysosomes are in yellow, mature lysosomes are in orange, lipofuscins are in pink, lipid bodies are in dark purple, myelinated axons are in blue, presynaptic axon terminals are in light green, and postsynaptic dendrites or dendritic spines are in dark green. Myelin degradation is distinguished by a white asterisk (*). Apparent cell bodies are identified by a capital letter, where “M” stands for microglia/microglial-like cell, and “A” stands for astrocytes. Scale bars are equivalent to 5 µm. (3–7) Graphs show means (thin wide bar) ± SEM, where individual values are represented by dark gray circles at day 0 (uninfected control) and black diamonds at day 6 p.i.

To provide additional functional insights into microglial involvement in HSV-1-infected VPL on day 6 p.i., we next performed ultrastructural analysis of microglia using nanoscale-resolution SEM. Microglia were identified based on their well-established distinctive ultrastructural characteristics [48]. As this method cannot fully differentiate between microglia and infiltrating monocytes, we refer to the examined cells as microglia/microglia-like cells, as described elsewhere [21]. Nanogold staining against HSV-1 (Fig. 2e-1) revealed that the virus was mostly located within endosomes containing digested contents (Fig. 2e-2) and at the cellular membrane of microglia/microglia-like cells (Fig. 2e-3), instead of being detected in the nucleus, where the majority of HSV-1 replication takes place [49]. This result supports the hypothesis that microglia/microglia-like cells phagocytose HSV-1^+^ cells to limit viral replication.

### Microglial functions are impaired in highly infected thalamic regions

We noticed that TMEM119 staining remained present in these regions despite the inflammatory environment [50]. These areas were populated by a mixture of hypertrophic and round-shaped microglia/microglia-like cells associated with a reactive state (Fig. 3a). Qualitative analysis of highly infected thalamus on day 6 p.i. showed a reduced hypertrophic/reactive microglial density. Most TMEM119^+^ ameboid microglia/microglia-like cells contacting the infected cells did not express CD68 (Figs. 2d, 3a). Despite decreased numbers of TMEM119^+^/CD68^+^ microglia/microglia-like cells, the highly infected thalamus was enriched in CD68^+^ puncta, suggesting that unknown microglial subsets downregulating TMEM119, or other phagocytes compensate for the impaired phagocytic activity of TMEM119^+^ microglia/microglia-like cells (Fig. 3a) [51, 52]. This CD68 expression was positively correlated with the intensity of the infection. Moreover, detecting HSV-1 signals on TMEM119^+^/CD68^−^ ramified microglia/microglia-like cells raised the intriguing possibility that these cells could be infected (Fig. 3a, right). Our results highlight an exacerbated expression of CD68, mainly found on other cells than TMEM119^+^ microglia/microglia-like cells in the highly infected thalamus.

**Fig. 4:**
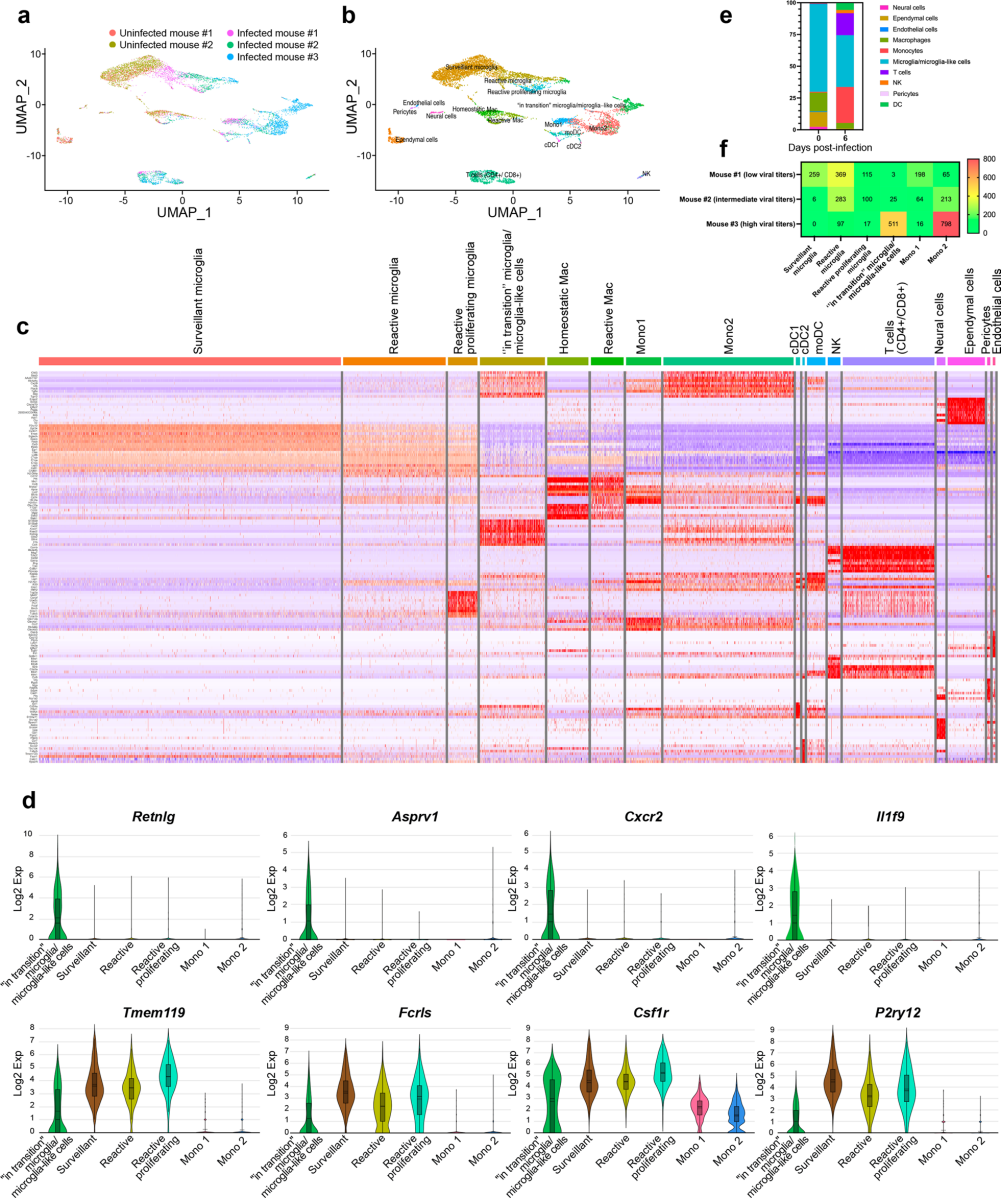
scRNA-seq reveals novel HSE-associated microglia/microglia-like cell transcriptome expressing neutrophil-related genes in highly infected thalamus. Chromium 10X coupled with Illumina sequencing was used to analyze the transcriptome of 2000 CD11b^+^ immune cells isolated with magnetic beads from the thalamus of the intranasally infected mouse on day 6 p.i. **a** td-UMAP visualization of aggregated scRNA-seq data, labeled by sample (three infected and two uninfected mice on day 6 p.i.), showing different cell clusters for each sample. **b** td-UMAP visualization of all sub-clusters labeled with different colors on aggregated scRNA-seq data. **c** Heatmap showing the top 150 genes whose levels of expression are highest and most differentiating in each of the 17 cell sub-clusters, revealing similar transcriptomic signatures between “in transition” microglia/microglia-like cell and Mono2 subsets. **d** Violin plots demonstrating differential expression (in log_2_ fold-change) of 4 neutrophils (*Retnlg*, *Asprv1*, *Cxcr2*, *Il1f9*) and 4 microglia (*Tmem119*, *Fclrs*, *Csf1r*, *P2ry12*)-related genes in “in transition” microglia/microglia-like cells, surveillant microglia, reactive microglia, reactive proliferating microglia, Mono1, and Mono2. Statistical analyses were performed on Loupe Browser v5. Statistically, significant results are indicated as follows: ****P < 0.001. **e** Stacked bar plot showing the mean relative proportion of each cell type in the thalamus of three HSV-1-infected mice (day 6 p.i.) and two uninfected control group (day 0). **f** Heatmap showing the number of surveillant, reactive, reactive proliferating microglia, “in transition” microglia/microglia-like cells, Mono 1 and Mono2 in three infected samples with different viral titers on day 6 p.i. The minimum and the maximum number of cells (0 and 800 cells, respectively) correspond to red and green colors, respectively

**Fig. 5:**
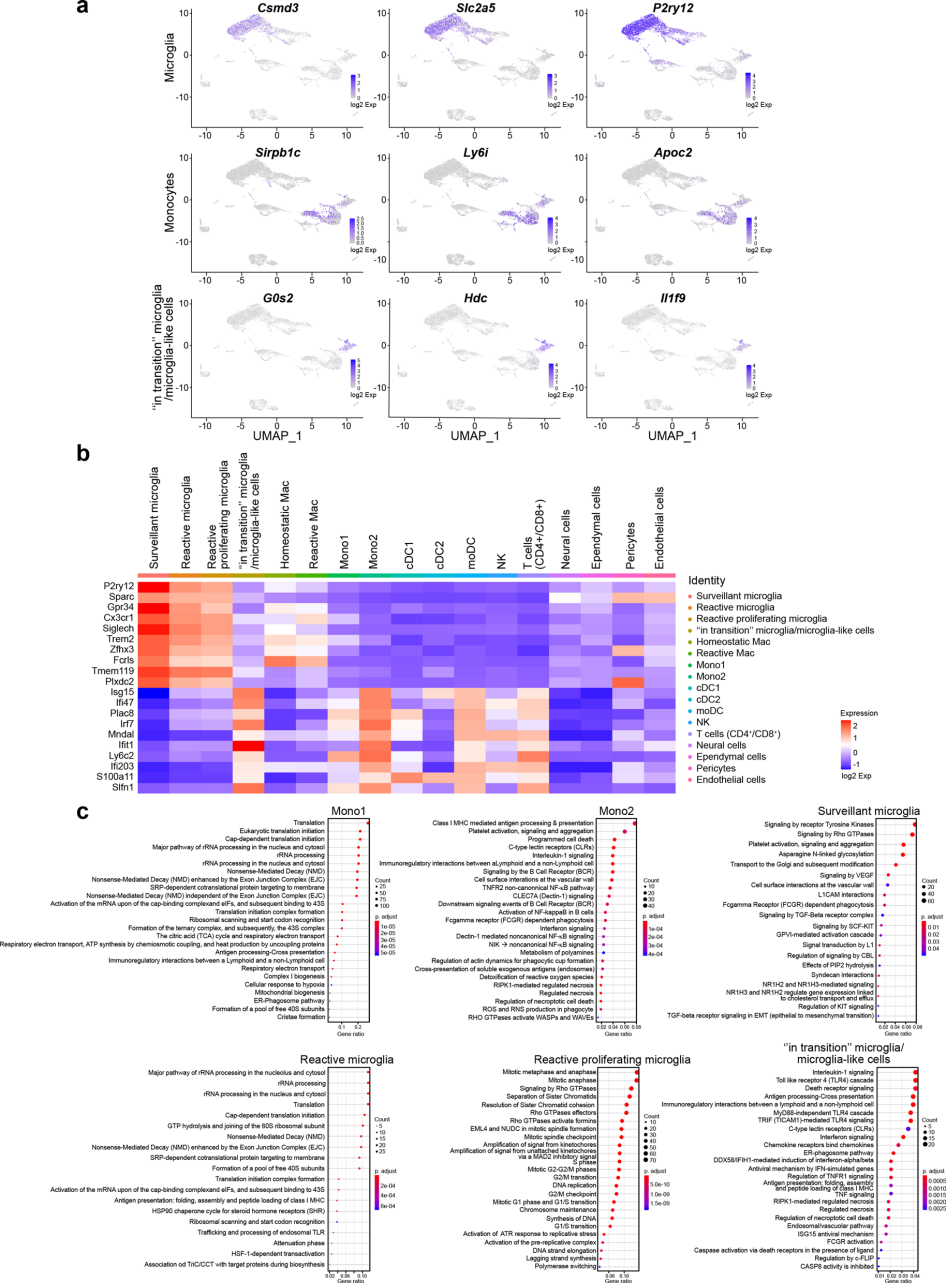
Analysis of up-regulated pathways uncovers HSV-1-mediated pro-inflammatory response of “in transition” microglia/microglia-like cells during HSE. **a** Feature plots show the distribution of the top three cluster-specific genes with the highest expression levels on two-dimensional UMAP (td-UMAP) visualization of aggregated data (three infected and two uninfected mice on day 6 p.i.). *Csmd3*, *Slc2a5*, *P2ry12* for microglia, *Sirpb1c*, *Ly6i*, *Apoc2* for infiltrating monocytes, and *G0s2*, *Il1f9*, *Hdc* for “in transition” microglia/microglia-like cells. **b** Homemade heatmap shows increased levels of IFN-stimulated genes involved in antiviral response for “in transition” microglia/microglia-like cells. **c** Dot plots showing the first 25 most significantly up-regulated Reactome pathways in monocytes and microglial cell sub-clusters. The size of each dot represents the number of genes in each cell sub-cluster, and the color of each dot indicates the normalized enrichment score of each pathway (p-adjust). Pathways that were not significantly enriched (*Q* value ≥ 0.05, Benjamini–Hochberg correction) are not displayed

**Fig. 6:**
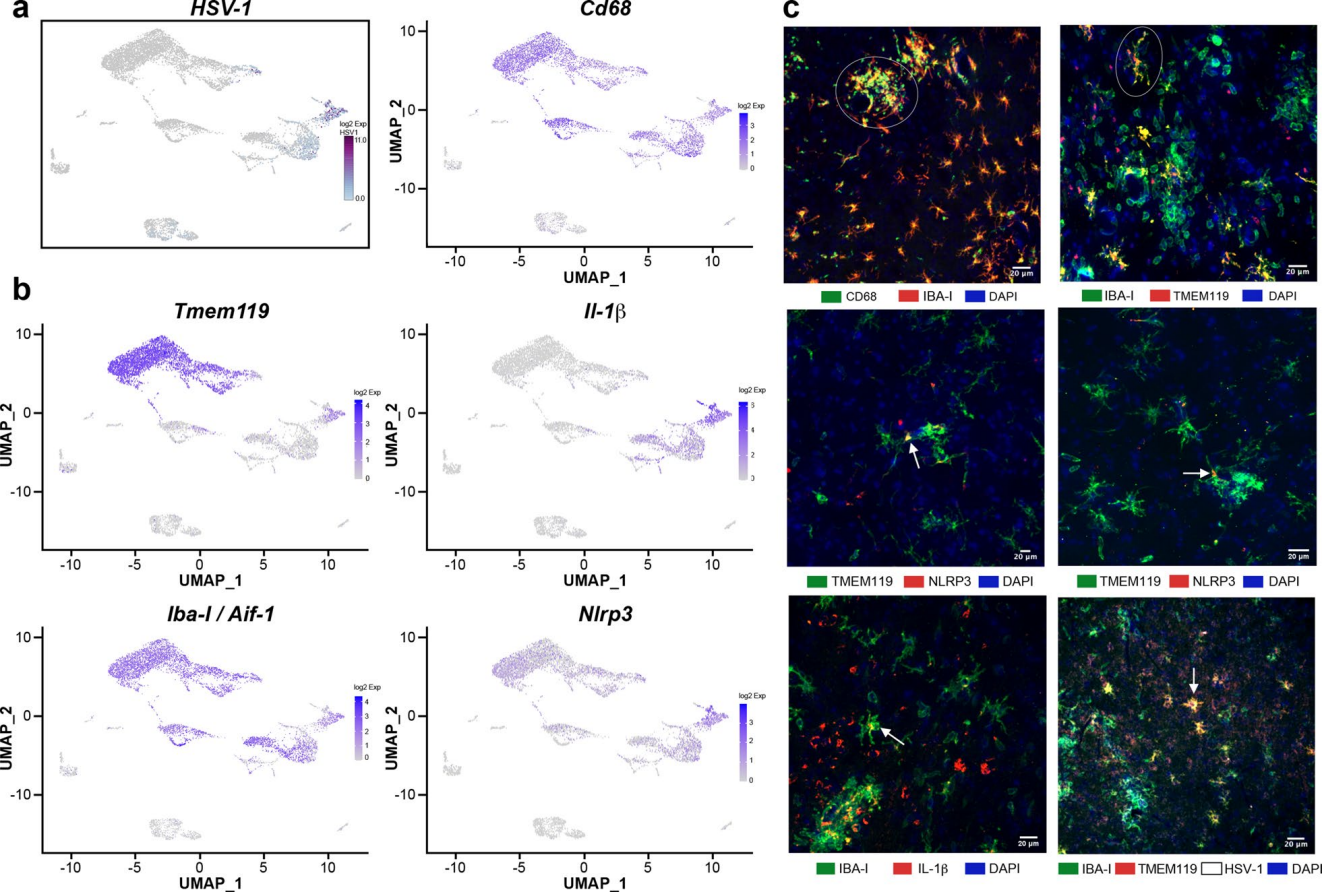
In situ characterization of “in transition” microglia/microglia-like cells in HSV-1-infected VPLs. **a** Distribution of HSV-1 transcripts was analyzed to identify infected cell sub-clusters on two-dimensional UMAP (td-UMAP) visualization of aggregated data (three infected and two uninfected mice on day 6 p.i.). “In transition” microglia/microglia-like cell sub-cluster corresponds to HSV-1-infected microglia/microglial-like cells. **b** Feature plots show the distribution of *Tmem119*, *Iba-I* (*Aif1*), *CD68*, *Nlrp3,* and *Il-1β* genes with on td-UMAP of aggregated data. Available antibodies against the proteins encoded by canonical genes were used to identify “in transition” microglia/microglia-like cells. **c** (top, left) IBA-I^+^ cells expressing high levels of CD68 clustered (white circle) in highly infected thalamus. (top, right) TMEM119 and IBA-I immunostaining revealed ramified TMEM119^+^/IBA-I^+^ cells (white circle) near clusters of TMEM119^−^/IBA-I^+^ cells in infected thalamus on day 6 p.i. Inflammasome activity in “in transition” microglia/microglia-like cells was studied using NLRP3 (middle, left, and right) and IL-1β (bottom, left) immunostaining on HSV-1^+^ VPLs. Confocal microscopy images revealed TMEM119^+^/NLRP3^+^ and IBA-I^+^/IL-1β ^+^ microglia/microglia-like cell populations (white arrow) highlighting “in transition” microglia/microglia-like cells. (bottom, right) Immunohistochemical staining for HSV-1 showed that these ramified TMEM119^+^/IBA-I^+^ cells were HSV-1^+^ (white arrow) (scale, 20 μm)

In parallel, ultrastructural analysis of microglia/microglia-like cells identified based on their ultrastructure (Fig. 3b-1, 2) was conducted by quantifying the number of organelles involved in phagolysosomal pathways on SEM images. This analysis revealed that microglia/microglia-like cells possessed a significantly reduced pool of immature lysosomes (also known as early endosomes) in the VPL on day 6 p.i. compared to day 0 (Fig. 3b-3, *P* < 0.05), whereas no significant change was seen for other organelles (Fig. 3b-4, 5; Additional file 1: Fig. S1, organelles). This observation supports the HSV-1-mediated disruption of microglial phagolysosomal pathways during HSE. Next, we evaluated the interactions that microglia/microglia-like cells established with their microenvironment, including CNS cells, such as other microglia/microglia-like cells, neurons, astrocytes, but also their subcellular compartments like myelinated axons, on the same SEM images (Additional file 1: Fig. S1, contacts). A reduced number of microglia/microglia-like cells made direct contact with myelinated axons (Fig. 3b-6, *P* < 0.01), but also with degraded myelin (Fig. 3b-7, *P* < 0.05) in the infected VPL. Taken together, we hypothesize that critical functions of microglia/microglia-like cells, such as the monitoring of neurons and clearance of myelin debris through phagocytosis, were impaired by a highly inflammatory environment in the HSV-1-infected thalamus.

### Identification of a novel microglia-like transcriptional signature in HSE

Our scRNA-seq analysis on CD11b^+^ cells isolated from the thalamus on day 6 p.i. provides a broad view of the transcriptional response of CD11b^+^ immune cells involved in the immune response during HSE. We mapped 7658 cells of an aggregated scRNA-seq data set consisting of five samples. Ten cell populations were identified based on UMAP and cell-specific markers (Additional file 2: Fig. S2a), i.e., T cells (*Cd3d*, *Trbc2*, *Trac*), natural killer cells (NK) (*Klra8*, *Klra4*, *Klrb1c*), monocytes (*Sirpb1c*, *Ly6i*, *Apoc2*), dendritic cells (DCs) (*Kmo*, H2-*DMb2*, *Ccr7*), macrophages (mac) (*Pf4*, *Mrc1*, *Cd163*), microglia (*P2ry12*, *Slc2a5*, *Sall3*), pericytes (*Vtn*, *Ndufa4l2*, *Pdgfrb*), ependymal cells (*Ttr*, *Enpp2*, *Ecrg4*), endothelial cells (*Ptprb*, *Ly6c1*, *Cldn5*) and neural cells (*Ptprz1*, *Gpr37l1*, *Ntsr2*).

Individually analyzed samples revealed that for each sample, major cell clusters were scattered differentially throughout the two-dimensional UMAP (td-UMAP) representation of aggregated scRNA-seq data (Fig. 4a). In parallel, viral titration of the remaining brain samples (brain without thalamus which was dissected for CD11b^+^ cell isolation) showed that each brain exhibited a different viral titer (Additional file 3: Fig. S3; Mouse #1: 57.000 PFUs/μL, Mouse #2: 98.000 PFUs/μL and Mouse #3: 136.000 PFUs/μL). We partitioned these immune cells into the following sub-clusters based on the expression of different genes, but also by taking into account different viral titers promoting different levels of inflammation: two monocytes (Mono1 = *Gm34084*, *Clec12a*, *Clec4a1*; Mono2 = *Ms4a8a*, *Arg1*, *Chil3*), two macrophages (reactive mac = *Ccl8*, *Cbr2*, *Cd163*; homeostatic mac = *Pf4*, *Cd209f*, *Mrc1*), three DCs (dendritic cells) (cDC1 (conventional DC) = *Xcr1*, *Snx22*, *Sept3*; cDC2 = *Cacnb3*, *Nudt17*, *Ccr7*; moDC (monocyte-derived dendritic cell) = (*Kmo*, *Ffar2*, *H2-DMb2*) and four microglia (surveillant microglia = *Csmd3*, *P2ry12*, *Gpr34*; reactive microglia = *C4b*, *Cacna1s*, *Hspa1b*; reactive proliferating microglia = *Cenpf*, *Pcr1*, *Top2a*; “in transition” microglia/microglia-like cells = *G0s2*, *Il1f9*, *Hdc*) (Additional file 3: Fig. S3). Thereafter, another td-UMAP was created to visualize these different cell sub-clusters on the aggregated data (Fig. 4b).

We generated a heatmap of the 150 most significantly up- and down-regulated genes to reveal distinct transcriptomic signatures of all sub-clusters. Overall, our clustering approach successfully distinguished the major cell populations and subpopulations with differential gene expression. As expected, we observed sub-clusters exhibiting similar gene expression patterns. Interestingly, Mono2 and “in transition” microglia/microglia-like cells shared a similar transcriptomic pattern, pointing out a possible differentiation between sub-clusters (Fig. 4c). Further analysis revealed 104 cells highly enriched (log_2_ fold-change > 8) in neutrophil-related genes. 79 of them belonged to “in transition” microglia/microglia-like cell sub-cluster (Additional file 2: Fig. S2b). Indeed, some well-known genes expressed by neutrophils such as *Retnlg*, *Asprv1*, *Il1f9,* and *Cxcr2* were also found in this novel microglia-like transcriptome [53]. We decided to define this cell subtype “microglia-like cells” considering that they highly expressed well-established microglial markers, such as *P2ry12*, *Tmem119*, *Fcrls,* and *Csf1r* (Fig. 4d). These findings suggest that the “in transition” microglia/microglia-like cell transcriptome whether corresponds to the transcriptomic signature of a new differentiating microglia-like sub-cluster or to microglia/microglia-like cells phagocyting infiltrating neutrophils [54, 55].

### The “in transition” microglia/microglia-like cell transcriptome is detected in the highly infected thalamus

We individually analyzed the kinetics of immune cells for each of the five scRNA-seq data sets. Two major clusters of uninfected thalami were microglia and mac, corresponding to 72.7% and 14% of all cell populations detected in those samples, respectively. We also noticed a decrease of microglia/microglia-like cells (72.7% vs. 40.4%, *P* 0.006) and CNS mac (14% vs. 3.3%, *P* 0.6529) percentages on day 6 p.i., compared to uninfected brains. By contrast, the levels of infiltrating monocytes significantly increased (0.6% vs. 30.6%, *P* 0.0073) on day 6 p.i. Monocytes (30.6%), T-cells (17.1%), DCs (4.5%), and NK cells (2.2%) were also present in the infected thalamus across the three examined samples, varying in their viral titers (Fig. 4e). In concordance with our flow cytometry data showing decreased numbers of microglia/microglia-like cells on day 6 p.i. (Fig. 2c), the analysis of cell population kinetics using scRNA-seq data also showed a decreased number of microglia/microglia-like cells on day 6 p.i.

Next, we evaluated the numbers of each microglial and monocytic sub-clusters for three infected samples. 259 surveillant and 369 reactive microglia were found in Mouse #1 sample with the lowest brain viral titer. In the same data set, we identified 115 reactive proliferating microglia and 3 “in transition” microglia/microglia-like cells. Mouse #2, with an intermediate viral titer, had 6 surveillant, 283 reactive, 100 proliferating reactive, and 25 “in transition” microglia/microglia-like cells. Reactive and reactive proliferating microglial sub-clusters, containing, respectively, 97 and 17 cells, continued to decrease with increased viral titers in Mouse #3 sample. The surveillant microglia sub-cluster completely disappeared, and “in transition” microglia/microglia-like cells reached the highest level with 511 cells in the same data set (Fig. 4f).

We observed high levels of infiltrating monocytes in all three infected mice differing in their viral titers. A total of 198, 64, 16 Mono1 and 65, 213, 798 Mono2 cells were detected in Mouse #1, #2, and #3 data sets, respectively. Mono2 levels increased with the severity of the infection (Fig. 4f). We also noticed that their numbers increased along with those of “in transition” microglia/microglia-like cells. This observation raised the question of whether those microglia/microglia-like cells differentiate from other microglial subsets, or Mono2 gives rise to this ambiguous population. Overall, these observations indicate that increased viral titers alter cell phenotypes and promote an increased “in transition” microglia/microglia-like cell transcriptome in the infected thalamus on day 6 p.i.

### The “in transition” microglia/microglia-like cell transcriptome indicates a hyperinflammatory microglial phenotype in highly infected thalamic regions

Monocytes and microglia represent hardly distinguishable cell populations in homeostatic conditions. Inflammation-driven phenotypic alterations of these cells in infectious disease models make their identification even more difficult. To distinguish microglial responses, we identified upregulated cluster-specific genes (log_2_ fold-change > 4; P < 0.05) for infiltrating monocytes, total microglia (surveillant, reactive and reactive proliferating), and “in transition” microglia/microglia-like cells. We included “in transition” microglia/microglia-like cells in this analysis as a major cluster, considering that the origin of these cells is still unknown. The top three most significantly upregulated cluster-specific genes for these three cell populations were: *Sirpb1c*, *Ly6i*, *Apoc2* for infiltrating monocytes, *Csmd3*, *P2ry12*, *Slc2a5* for microglia and *G0s2*, *Il1f9*, *Hdc* for “in transition” microglia/microglia-like cells (Fig. 5a (Top 3 genes); Additional file 2: Fig. S2c (Top 50 genes). This analysis allowed the identification of potential biomarkers that could be used to differentiate these clusters.

Furthermore, we performed Panther—Gene Ontology (GO) Enrichment Analysis for molecular functions on “in transition” microglia/microglia-like cell cluster, using all significantly up-regulated genes (P < 0.05). The analysis revealed the following upregulated GO terms: “Protein homodimerization activity,” “Chemokine receptor binding,” “Cytokine binding,” “Cytokine activity,” “Carbohydrate derivative binding,” “Nucleotide binding,” “GTPase activity,” and “Enzyme binding.” Interestingly, “double-stranded RNA binding” function, regrouping 6 genes (*Ifih1*, *Adar*, *Oas2*, *Oas3*, *Oasl1,* and *Oasl2*) involved in the antiviral response, including IFN downstream signaling, were identified for this microglial sub-cluster [56–58]. Indeed, the assessment of differential expression levels of a homemade gene set demonstrated upregulated expression levels of genes encoding antiviral proteins and IFN-related genes, such as *Isg15*, *Ifi47*, *Irf7*, *Ifit1*, for the “in transition” microglia/microglia-like cells (Fig. 5b). These results suggest a direct antiviral response orchestrated by “in transition” microglia/microglia-like cells during HSE.

We performed an exploratory study of biological pathways enriched in infiltrating monocytes and microglial sub-clusters to assess cellular functions using the Reactome Knowledgebase (Fig. 5c) (Additional file 4: Fig. S4 Reactome pathway dot plots and enrichment maps for Mono2 and “in transition” microglia/microglia-like cells) (Additional file 5: Fig. S5 Functional heat plots for Reactome pathway gene sets) [59]. Infiltrating Mono1 up-regulated genes associated to metabolic pathways (e.g.,* Atpc1*, *Eif3f*, *Ndufa2*), “Antigen processing-Cross presentation (e.g., *Cyba*, *H2-M3*, *Psma2*) and “Immunoregulatory interactions between a Lymphoid and non-Lymphoid cell” (e.g., *B2m*, *H2-T22*, *Fcgr2*) functions, suggesting an increased capacity for antigen processing and presentation (Additional file 4: Fig. S4a, e; Additional file 5: Fig. S5a). Mono2 appeared to be one of the main cytokine/chemokine producers (e.g., *CXCL2/10*, *IL-1α/β*, *IL-15/18/23a/27*) during HSE. We noticed Mono2 activated CLEC7A signaling, which plays a role in the production of TNF, CXCL2, and IL-1β [60]. Amongst several other pathways, “Class I MHC-mediated antigen processing & presentation,” “C-type lectin receptors” (e.g., *C3*, *Clec4n*, Fcer1g), and “Fc gamma receptor (FCGR) dependent phagocytosis” (e.g., *Actb*, *Arpc3*, *Fcgr4*, etc.), were up-regulated in Mono2. We also detected upregulation of the “TNFR2 non-canonical NF-KB pathway” (e.g., *Birc3*, *Cd40*, *Nfkb2*) and “Programmed cell death” (e.g., *Bax*, *Bcl2l1*, *Casp3*) for these cells on day 6 p.i. (Fig. 5c, top; Additional file 5: Fig. S5b).

Surveillant microglia were enriched in “Signaling by Receptor Tyrosine Kinases” (e.g., *Col27a1*, *Dusp7*, *Insr*), “Signaling by Rho GTPase” (e.g., *Fgd2*, *Rhoq*, *Tuba1a*), and “Platelet activation, signaling and aggregation” (e.g., *Gna12*, *Mapk14*, *Pic3cg*), “Signaling by VEGF” (e.g., *Ncf2*, *Prkar1a*, *Rock2*) that activates angiogenesis and “Signaling by TGF-beta Receptor Complex” with immunosuppressive functions (Additional file 4: Fig. S4a; Additional file 5: Fig. S5c). In addition, increased expression of the “FCGR dependent phagocytosis” gene set suggested that surveillant microglia exhibit phagocytic activity while maintaining CNS homeostasis. Reactive microglia activated similar RNA and protein metabolic pathways as found in infiltrating Mono1, e.g., rRNA processing (e.g., *Ddx21*, *Gnl3*, *Nop56*), translation (e.g., *Pabpc1*, *Eef1g, Rps8*), and non-mediated decay (NMD) (e.g., *Rpl11*, *Rpl12*, *Rpl14*). These cells increased the expression of genes encoding heat–shock proteins (HSPs) (e.g., *Hsp90aa1*, *Hsp90ab1*, *Hspa1a*) involved in the control of oxidative stress, inflammatory response, and antigen presentation [61]. Moreover, Endosomal TLR-mediated recognition of HSV-1 seemed to activate these phagocytes that also play a major role in antigen presentation via the major histocompatibility complex (MHC)-I, but also MHC-II (Additional file 4: Fig. S4c; Additional file 5: Fig. S5d). These results suggest that reactive microglia directly affect CD4^+^ T cell response [62]. Our results also confirm that microglia proliferate in the CNS upon viral infection. For these reactive proliferating microglia, we identified mitotic cell division pathways, such as “Mitotic Anaphase and Metaphase” (e.g., *Banf1*, *Birc5*, *Anapc5*), “Separation of sister chromatids” (e.g., *Bub1*, *Bub1b*, *Cdc20*), “DNA replication” (e.g., *Dbf4*, *E2f2*, *Gmnn*) and “G2/M checkpoints” (e.g., *Cdk1*, *Exo1*, *Herc2*). We suggest that microgliosis can contribute to the repopulation of these empty niches caused by reactive or dying microglia in the thalamus, although these proliferating microglia exhibiting pro-inflammatory features could also be responsible for the aggravation of the immune response during HSE (Additional file 4: Fig. S4d; Additional file 5: Fig. S5e).

The analysis of “in transition” microglia/microglia-like cells showed that the most significantly enriched pathway was “Interleukin-1 signaling” (e.g., *Il1a*, *Il1b*, *Il1r2*). These microglia/microglia-like cells were activated via multiple TLRs (*Tlr2/3/4/6/7*). Based on increased expression of *Tram*, *Trif,* and the other genes involved in downstream signaling of TLR4, “TRIF-mediated TLR4 signaling” (e.g., *Birc3*, *Cd14*, *Ikbke*) came up as the major pathway. However, we believe that not only TLR4 but also a multitude of TLRs led to the induction of a hyperinflammatory phenotype producing a variety of cytokines/chemokines exacerbating the inflammatory response [63]. This microglial sub-cluster increased the expression of the transcription factor AP-1 (*Jun*), Interferon Regulatory Factors (*Irf1/7/8*), as well as other genes (*Jak1*, *Stat1*, *Stat2*, *cGas*, *Tmem173* (*Sting*), etc.) involved in the IFN-induced JAK–STAT pathway (Fig. 5c, bottom; Additional file 4: Fig. S4f; Additional file 5: Fig. S5f). Our results confirm the up-regulation of the “Interferon signaling” (e.g., *Eif2ak2*, *RnaseI*, *Socs3*) pathway, suggesting that these microglia/microglia-like cells play a major role in viral detection and type I IFN production.

“ER-Phagosome pathways” (e.g., *H2-Q10*, *H2-T23*, *B2m*) and “Antigen-presentation via MHC-I” (e.g., *H2-Q6*, *Tap1*, *Tap2*) were activated in “in transition” microglia/microglia-like cells, as well as in reactive microglia and Mono2. Contrarily to other microglia and monocyte sub-clusters, antiviral pathways such as “Antiviral mechanism by IFN-stimulated genes” (e.g., *Arih1*, *Usp18*, *Oasl1*), “DDX58/IFIH1-mediated induction of interferon-alpha/beta” (e.g., *Nfkb1*, *Nfkb2*, *Tax1bp1*), and “ISG15 antiviral mechanism” (e.g., *Isg15*, *Trim25*, *Ube2l6*) were present in the pathway list of “in transition” microglia/microglia-like cells (Fig. 5c). They overexpressed dsRNA helicase genes [e.g., *Mda5* (*Ifih*), *Lgp2* (*Dhx58*)], Interferon Stimulated Exonuclease Genes (e.g., *Isg15* and *Isg20*) and other genes encoding antiviral proteins like *Acod1* (Aconitate Decarboxylase 1), *Ifit1* (Interferon-induced protein with tetratricopeptide repeats 1 Gene), *Rsad2* (radical SAM domain-containing, also known as Viperin) and *Hcar2* (Hydroxycarboxylic Acid Receptor 2) [57, 64–68]. Based on these highly expressed cytoplasmic antiviral factors, we hypothesize that “in transition” microglia/microglia-like cells with hyperinflammatory features correspond to HSV-1-infected microglia attempting to inhibit viral replication.

We also observed significant enrichment of programmed cell death pathways, including “RIPK1-mediated regulated necrosis” (e.g., *Cflar*, *Fas*, *Mlkl*), “Regulated Necrosis” (e.g., *Peli1*, *Ripk1*, *Sdcbp*), “Regulation of necroptotic cell death” (e.g., *Birc3*, *Casp8*, *Ube2l6*), for “in transition” microglia/microglia-like cells. In parallel, overexpression of genes (e.g., *Ripk3*, *Fas*, *Tnf*, *Tnfsf10*, *Nlrp3*) involved in TNF and NLR signaling were observed. Overexpressed “casp8 activity is inhibited” in “in transition” microglia/microglia-like cells, suggesting that the blockage of CASP8 activity in the presence of viral FLIP-like protein switches apoptotic signaling to necrotic cell death [69]. In addition, higher expression of *Cflar* (cellular FADD-like interleukin-1 beta converting enzyme (FLICE)-inhibitory protein (cFLIP, encoded by the *Cflar*)) inhibiting apoptosis and promoting necroptosis, strengthens the possibility that “in transition” microglia/microglia-like cells undergo necroptosis.

### The “in transition” microglia/microglia-like cell transcriptome corresponds to the antiviral response of HSV-1-infected microglia

To clarify whether “in transition” microglia/microglia-like cells phagocytose-infected cells or represent HSV-1-infected microglia, we searched for reads corresponding to the viral transcripts in our scRNA-seq data as described previously [70]. Our data showed that few Mono1 and T cells contained viral mRNA. We noticed that Mono2 was highly enriched in viral transcripts. Of note, “in transition” microglia/microglia-like cells exhibited the most elevated levels of HSV-1 transcripts (log_2_ fold-change > 5, Fig. 6a). We suggest that similar transcriptomes of “in transition” microglia/microglia-like cells and Mono2 could result from HSV-1 infection activating similar antiviral mechanisms. Altogether, such high levels of viral transcripts suggest a potential viral replication phase for these two clusters.

Furthermore, we characterized HSV-1-infected “in transition” microglia/microglia-like cells by immunofluorescence and confocal microscopy. First, we confirmed that this sub-cluster expressed *Tmem119*, *Iba-I* (*Aif1*), *CD68*, *IL-1β,* and *Nlrp3* (Fig. 6b). IF images revealed that IBA-1^+^/CD68^+^ cells forming clusters became frequent towards highly infected spots, while the number of TMEM119^+^/CD68^+^ cells gradually decreased (Fig. 6c, top-left). We further observed ramified TMEM119^+^/IBA-I^+^ microglia/microglia-like cells in the vicinity of highly infected spots (Fig. 6c, top-right). In line with transcriptomic results, highly infected spots in the thalamus accommodated TMEM119^+^/NLRP3^+^, and IBA-I ^+^/IL-1β^+^ ramified cells that potentially correspond to “in transition” microglia/microglia-like cells (Fig. 6c, both middle and bottom-left). In addition, the TMEM119^+^/IBA-I^+^ cells were also HSV-1^+^ (Fig. 6c, bottom-right). These overall observations suggest that the few TMEM119^+^/IBA-I^+^ ramified cells localized in highly infected regions of the thalamus correspond to “in transition” microglia/microglia-like cells.

## Discussion

HSE is a rare neurological disorder characterized by CNS inflammation. Numerous cell types, including CNS resident cells and peripheral immune cells, which participate in the immune response against HSV-1, make HSE a complex disease to decipher. Previous studies showed that an early innate immune response has a critical role in controlling HSV-1 infection of the brain [14]. However, this early reaction progresses toward an uncontrolled inflammation, considered harmful, in the late stage of HSE. Microglia, the first immune cell to respond against the infection, represents a potential target for therapeutic interventions. Despite various studies on neuroinfectious diseases, little is known about their roles in HSE. This study sought insights into how microglia react to HSV-1 infection, using a single-cell sequencing approach combined with different complementary microscopy methods.

We first characterized our intranasally infected HSE mouse model. Following the observation of neurological symptoms, HSE-associated deaths started to occur on day 6 p.i. Further analysis demonstrated that the hindbrain and thalamus were two highly infected regions on day 6 p.i. The VPL was identified as the most infected area of the CNS during the peak of HSE. We studied the microglia/microglia-like cells within highly infected thalamic regions to better understand their contribution to this exacerbated immune response. Our findings reveal that HSV-1-infected microglia/microglia-like cells detect viral presence using cytosolic DNA/RNA sensors, then adopt a hyperactive phenotype producing IL-1β and TNF-α. These neurotoxic microglia, so-called “in transition” microglia/microglia-like cells found in highly infected thalamus, could accelerate the proinflammatory response and worsen the outcome of HSE.

It is commonly accepted that HSV-1 uses the olfactory nerve to infiltrate the brain following intranasal inoculation. The low detection of HSV-1^+^ cells in olfactory bulbs and tubercles demonstrated that the olfactory route remains a potential pathway for HSV-1 to access the CNS. Unexpectedly, the hindbrain exhibited a more intense bioluminescent signal compared to the olfactory bulbs. This observation suggests that thalamic infection could occur earlier than in other regions, causing higher viral titers. These results also indicate that the neuroepithelium may not be the only route to the CNS for intranasally inoculated HSV-1. Moreover, the observation of high titers in the VPL, known to be connected to the brainstem trigeminal complex via ventral trigeminal tract major, strengthens the idea that HSV-1 enters the CNS via the projections of trigeminal nerves [71, 72]. Taken together, these findings indicate that HSV-1 infiltrates the CNS through multiple ways in our HSE model.

Besides viral replication, HSE is characterized by an important number of infiltrating peripheral immune cells among the CNS. On day 6 p.i., similar percentages of CD45^high^ infiltrating cells and CD45^low/inter^ microglia/microglia-like cells in whole brains highlighted the contribution of infiltrating immune cells. Increased CD45 expression in microglia has already been detected in several CNS pathologies, such as Alzheimer’s disease and multiple sclerosis [73]. Our scRNA-seq analysis confirmed that infiltrating immune cells expressed similar Ptprc (CD45) levels within all microglial subsets, except for the surveillant microglia found before the infection. Altogether, we think that CD45^high^ CD11b^+^ gating containing phenotypically altered CD45^high^ microglia/microglia-like cells on day 6 p.i. may overestimate the numbers of CD45^+^ infiltrating leukocytes. Monocytes, sharing common proprieties with microglia, represented one of the two major infiltrating immune cell populations on day 6 p.i. The abundant infiltration of monocytes in the CNS complicated the analysis of microglial response, thus highlighting the importance of identifying cell-specific genes. CD11b^+^CD45^+^Ly6C^+^ gating was used to identify monocyte with flow cytometry. However, we noticed that *Ly6c* was also expressed by “in transition” microglia/microglia-like cells, suggesting that this gating identifies more than monocytes. Highly expressed *CD45* and *Ly6c* by this new microglial sub-cluster highlighted that (1) each marker used to identify an immune cell by flow cytometry should be well characterized and (2) new cell-specific markers are required to better identify immune cells. Based on our findings, *Sirpb1c*, *Ly6i,* and *Apoc2* represent candidate monocyte-specific markers, whereas *Csmd3*, *P2ry12,* and *Slc2a5* are microglia-specific in the context of HSE.

Microglia exert an antiviral effect during CNS infection through their phagocytosis capacity and production of cytokines/chemokines that include type I IFN. Previous studies have shown that the phagocytic uptake of infected cells by reactive microglia is crucial for controlling viral spread [74, 75]. Another study reported that microglial depletion resulted in ineffective T cell responses during lethal encephalitis caused by a neurotropic coronavirus [76]. In HSE, their absence was associated with a decreased expression of IFN-β and reduced numbers of monocytes and T cells infiltrating the CNS, worsening the outcome of HSE [14]. In agreement with these previous studies, our overall analysis demonstrated that TMEM119^+^/CD68^+^ reactive microglia/microglia-like cells were able to fulfill their main functions, such as phagocytosis and antigen presentation, in the thalamus of mice with low viral loads.

The analysis of highly infected thalamic regions further revealed an increased proportion of ameboid microglia/microglia-like cells found in highly inflammatory environments. Reduced microglial interactions with nodes of Ranvier seems to promote a pro-inflammatory phenotype in spinal cord injury [77]. We also demonstrated that HSE-associated microglia/microglia-like cells decreased their number of contacts with myelinated axons and degraded myelin, supporting the potential acquisition of a pro-inflammatory phenotype. Contrarily to microglia found in most viral encephalitis, these extremely reactive microglia lost CD68 expression among regions with high viral loads [78]. In line with the low levels of CD68 expression in reactive microglia, ultrastructural analysis of the VPL revealed reduced numbers of immature lysosomes in microglia/microglia-like cells, indicating disrupted phagolysosome pathways. The decrease in immature lysosomes, already reported in neurodegenerative pathologies, may also be explained by the potential fusion of these structures within the Golgi compartment during viral assembly, suggesting that these microglia/microglia-like cells are infected with HSV-1 [79]. Interestingly, we observed an important number of CD68^+^ puncta located outside microglia/microglia-like cells in the highly infected thalamus, emphasizing the involvement of other phagocytes compensating for impaired microglial phagocytosis [51, 80]. Taken together, these findings suggest that the exacerbated inflammatory response in the highly infected thalamus impaired microglial functions. At the same time, microglia/microglia-like cells exhibited a protective role in the moderately infected thalamus.

The transcriptomic signatures of immune cells, including microglia, were assessed using a single-cell sequencing approach. Four microglial sub-clusters were identified from our aggregated scRNA-seq data. Surveillant, reactive, and reactive proliferating microglia expressing microglial signature genes were located close to one another on the td-UMAP distribution. Surprisingly, we observed another cell cluster closer to Mono2, which only appeared in the infected samples. The frequency of these cells increased progressively with the viral titers, indicating their association with the severity of infection. We conclude that these cells arose from microglia, considering their high expression levels of microglial signature genes. They were thus named “in transition” microglia/microglia-like cells. We initially thought that this new population connecting the transcriptomes of both Mono2 and reactive microglia might correspond to a new transition state of infiltrating monocytes differentiating into microglia-like cells. The simultaneous increase of infiltrating monocytes and decrease of CD45^low/inter^ microglia/microglia-like cells strengthens the hypothesis that microglia acquire a Mono2-like transcriptome. Monocyte-to-microglia/microglia-like cells transition was studied in various CNS pathologies, in which monocytes become microglia-like cells [20]. Some studies claimed that during viral encephalitis, infiltrating Ly6C^+^ monocytes do not give rise to microglia [24]. Further investigations are needed to understand if the “in transition” microglia/microglia-like cell represents a transitional Mono2/microglia phenotype in HSE. Based on our observations, we suggest that HSV-1 infection results in a convergent transcriptional response for infiltrating monocytes and resident microglia. Taken together, we conclude that “in transition” microglia/microglia-like cells correspond to an antiviral state.

To better understand their role in HSE, we next investigated molecular pathways of microglia/microglia-like cells and monocytes. Reactive microglia up-regulated nonsense-mediated mRNA decay pathway involved in the degradation of viral mRNAs. The viral replication modulating effect of the NMD pathway has already been demonstrated in murine hepatitis virus and Kaposi’s sarcoma-associated herpesvirus infections [81, 82]. These reactive microglia also actively contributed to antigen presentation. Conversely, Mono1 expressing *H2-Eb1*, *H2-Aa,* and *H2-Ab1* may be more crucial in triggering adaptive responses than reactive microglia. These findings raise the intriguing possibility that CCR2^+^ Mono1 infiltrated the infected CNS, then acquired a Mono2 phenotype with increased antiviral features by up-regulating genes encoding cytokines/chemokines and other antiviral effectors [83]. These cells represent one of the two HSV-1 mRNA enriched cell types. Further analysis suggested that “in transition” microglia/microglia-like cells were infected by HSV-1. In addition to the detection of viral mRNA in these cells, IF analysis showed that HSV-1 antibodies stained the entire cell instead of phagosomes containing infected cell debris [14, 78]. Overall, gene profiling indicates that “in transition” microglia/microglia-like cells detect the dsRNA of HSV-1 via RIG-I (retinoic acid-inducible gene I, also known as DDX58)/MDA5 in the cytosol, resulting in type I IFN production and NLRP3 inflammasome-mediated IL-1β secretion [84, 85]. In concordance with an increased pro-inflammatory profile, accelerated microglial exosome release may result in decreased early endosome numbers in reactive microglia during HSE [86]. These pro-inflammatory TMEM119^+^/IBA-I^+^ “in transition” microglia/microglia-like cells can activate NLRP3-inflammasome-mediated IL-1β production in the infected thalamus. A recent study revealed that inflammasome response to HSV is primarily mediated by microglia and contributes to mortality independent of controlling viral replication [87]. Similar to our novel HSE-associated microglial transcriptomic findings, a novel subset of high-grade glioma-associated microglia exhibiting inflammasome-mediated pro-inflammatory and proliferative signatures has been recently described [88]. Another study reported that neurotoxic CCR9^+^ACOD1^+^ microglia producing high levels of TNF-α was observed in the CNS of mice infected with neurotrophic pathogens. As observed in “in transition” microglia/microglia-like cells, highly expressed *Acod1* was associated with neurotoxic microglia, which were resistant to apoptosis and associated with chronic neuroinflammation [66, 89]. “In transition” microglia/microglia-like cells exhibited a swollen but ramified morphology, called hypertrophic, contrary to other hyperactive ameboid microglia/microglia-like cells found in the highly infected thalamus. In accordance with the cytoplasmic swelling observed during necroptosis, pathway analysis also suggested the activation of TNF signaling and necroptotic cell death [90, 91]. Activated programmed cell death pathways in “in transition” microglia/microglia-like cells could explain the up-regulation of neutrophil-related genes. Further studies are required to clarify whether “in transition” microglia/microglia-like cells undergo necroptosis or are involved in the phagocytic removal of necroptotic neutrophils to prevent them from releasing their cytotoxic content [92]. Following the exposure to strong immune-stimulatory signals, microglia/microglia-like cells undergo apoptotic cell death to preserve brain tissue from the damaging effect caused by long-term activation of these cells in the HSV-1-infected brain [93]. However, viral RIR1 interaction with RIPK1, RIPK3, and CASP8 seemed to block apoptosis and prevented TNF-induced necroptosis in infected human cells [93–95]. We suggest that blocking cell death by HSV-1 infection could result in a persistent “in transition” microglia/microglia-like state producing pro-inflammatory mediators [96].

In summary, we showed that although microglia/microglia-like cells could exert a beneficial role in the early stage of HSV-1 infection, the same cells could alter their functions and have detrimental effects throughout HSE [97]. Our study uncovered a novel transcriptional response of microglia/microglia-like cells during HSE while exploring the functions exhibited by different microglial states. Nonetheless, our study has some limitations. To validate that HSV-1 RNA and proteins detected “in transition” microglia/microglia-like cells, are originated from a productive infection and not from phagocytosed HSV-1^+^ cells, another approach like single nucleus RNA sequencing should be performed. Our conclusions about the morphology, density, and distribution of microglia/microglia-like cells and other immune cells should be carefully interpreted, since we did not perform a quantitative analysis of IF images. Another limitation is the low number of samples for scRNA-seq, which could mislead the interpretations of our transcriptomic data. Further studies with larger number of mice and timepoints would allow to confirm our observations. We could also not compare our RNA sequencing results between the three infected samples, initially designed as biological replicates because of differences in brain viral titers. On the other side, analyzing microglia/microglia-like cells exposed to different viral loads allowed us to study diverse states of these cells and to characterize transcriptomic responses of infected “in transition” microglia/microglia-like cells. Our RNA-seq data provide a resource for future investigations of the roles of microglia/microglia-like cells in HSE.

## Conclusions

We suggest that distinct “in transition” microglia/microglia-like cells represent an important player leading to an exacerbated pro-inflammatory response and could thus become a promising target to modulate neuroinflammation.

## Supplementary Information

**Additional file 1: Figure S1.** Microglial and microglial-like cell organelles and elements of their microenvironment that are in contact with the cell body were analyzed on SEM images of VPL from days 0 to 6 p.i. Graphs show means (thin wide bar) ± SEM, where individual values are represented by dark gray circles at day 0 (uninfected control) and black diamonds at day 6 p.i.

**Additional file 2: Figure S2.** UMAP visualization (a) of aggregated and individual scRNA-seq data of two uninfected and three infected mice on day 6 p.i., showing different major cell clusters for each sample. (b) UMAP visualization of cells enriched in neutrophil-related genes on Loupe Browser (log_2_ fold-change > 8 for *Il1a*, *Ccl2*, *Ccl3*, *Tnf*, *Cxcl10, Cxcr4 together*). (c) The analysis of upregulated cluster-specific genes (log_2_ fold-change > 4; P < 0.05) for infiltrating monocytes, total microglia (surveillant, reactive and reactive proliferating), and “in transition” microglia/microglia-like cells. “In transition” microglia/microglia-like cells were considered a major cell cluster. Major clusters were analyzed for locally distinguishing genes on Loupe Browser.

**Additional file 3: Figure S3.** UMAP visualization of aggregated and individual scRNA-seq data of two uninfected and three infected mice on day 6 p.i., showing different cell sub-clusters for each individual sample. Representative plaque assay wells used to determine the viral titers are provided below UMAP.

**Additional file 4: Figure S4.** Enrichment maps of Reactome pathways for Mono2 (a) and “in transition” microglia/microglia-like cells (b). The size of each dot represents the number of genes in each cell sub-cluster, and the color of each dot indicates the normalized enrichment score of each pathway (p-adjust). Pathways that were not significantly enriched (Q value ≥ 0.05, Benjamini–Hochberg correction) are not displayed.

**Additional file 5: Figure S5.** Heatplots for monocytes and microglial cell sub-clusters. Heatmap-like functional classification of different genes into Reactome pathways were performed for (a) Mono1, (b) Mono2, (c) surveillant, (d) reactive, (e) reactive proliferating, and (f) “in transition” microglia/microglia-like cells.

## Data Availability

The scRNA-seq data sets and SEM images that support the findings of this study are available upon reasonable request from the corresponding author.

## Abbreviations

BH: Benjamini–Hochberg method
cDC: Conventional dendritic cell
CNS: Central nervous system
DC: Dendritic cell
DPBS: Dulbecco’s phosphate-buffered saline
EDTA: Ethylenediaminetetraacetic acid
GEM-RT: Gel beads-in-emulsion reverse transcription
GEM: Gel beads-in-emulsion
GO: Gene ontology
HSE: Herpes simplex encephalitis
HSP: Heat–shock protein
HSV-1: Herpes simplex virus 1
IF: Immunofluorescence
IFN: Interferon
IRF: Interferon regulatory factors
IVIS: Intravital imaging system
KPBS: Potassium-phosphate buffered saline
Mac: Macrophage
MACS: Magnetic-activated cell sorting
MHC: Major histocompatibility complex
Mono: Monocyte
NK: Natural killer
NMD: Non-mediated decay
NO: Nitric oxide
p.i.: Post-infection
PB: Phosphate buffer
PBS: Phosphate-buffered saline
pDC: Plasmacytoid dendritic cells
PFA: Paraformaldehyde
rHSV-1: Recombinant herpes simplex virus 1
ROS: Reactive oxygen species
RT: Room temperature
scRNA-seq: Single-cell RNA sequencing
SEM: Scanning electronic microscopy
td-UMAP: Two-dimensional uniform manifold approximation and projection
tSNE: T-distributed stochastic neighbor embedding
UMAP: Uniform manifold approximation and projection
VPL: Ventral posterolateral nucleus
VPN: Ventral posterior nucleus
