# Supplementary figures and images for: Single-cell transcriptomics of the ventral posterolateral nucleus-enriched thalamic regions from HSV-1-infected mice reveal a novel microglia/microglia-like transcriptional response
Source: J Neuroinflammation. 2022 Apr 6;19:81. doi: 10.1186/s12974-022-02437-7 (PMC8985399; doi:10.1186/s12974-022-02437-7)

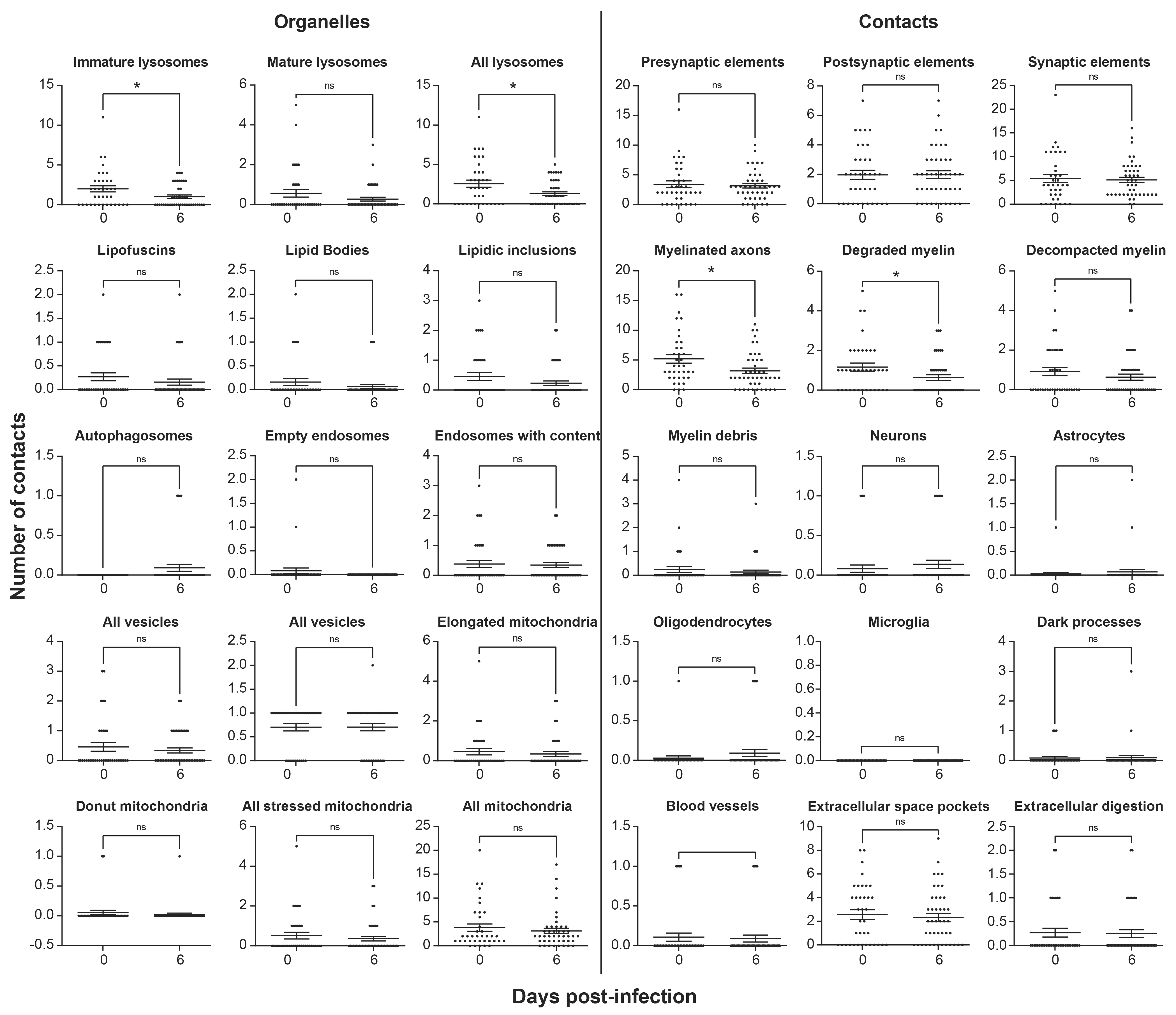

Supplement: Supplementary file 1 — Additional file 1: Figure S1. Microglial and microglial-like cell organelles and elements of their microenvironment that are in contact with the cell body were analyzed on SEM images of VPL from days 0 to 6 p.i. Graphs show means (thin wide bar) ± SEM, where individual values are represented by dark gray circles at day 0 (uninfected control) and black diamonds at day 6 p.i. [file 12974_2022_2437_MOESM1_ESM.tif]

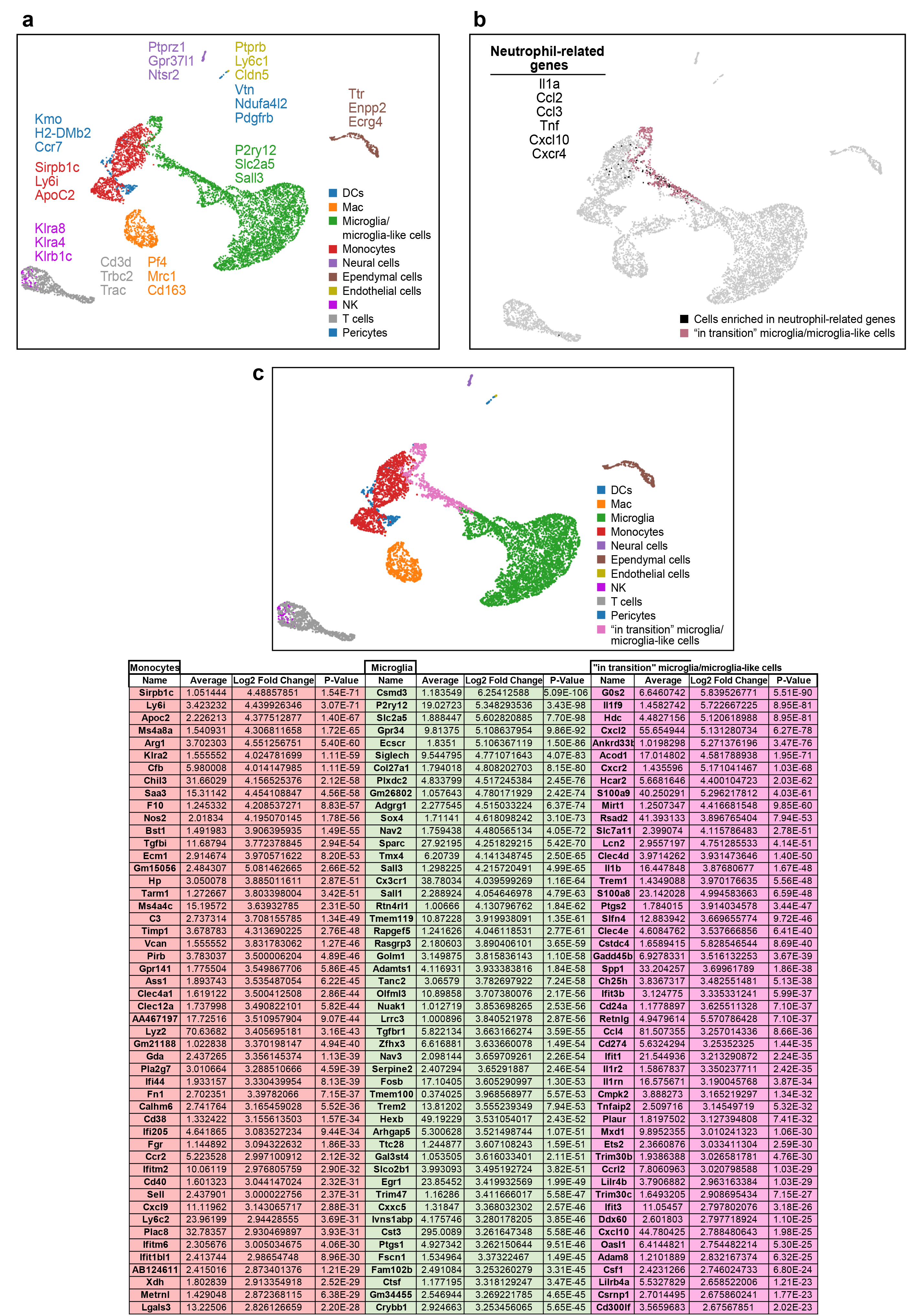

Supplement: Supplementary file 2 — Additional file 2: Figure S2. UMAP visualization (a) of aggregated and individual scRNA-seq data of two uninfected and three infected mice on day 6 p.i., showing different major cell clusters for each sample. (b) UMAP visualization of cells enriched in neutrophil-related genes on Loupe Browser (log2 fold-change > 8 for Il1a, Ccl2, Ccl3, Tnf, Cxcl10, Cxcr4 together). (c) The analysis of upregulated cluster-specific genes (log2 fold-change > 4; P < 0.05) for infiltrating monocytes, total microglia (surveillant, reactive and reactive proliferating), and “in transition” microglia/microglia-like cells. “In transition” microglia/microglia-like cells were considered a major cell cluster. Major clusters were analyzed for locally distinguishing genes on Loupe Browser. [file 12974_2022_2437_MOESM2_ESM.tif]

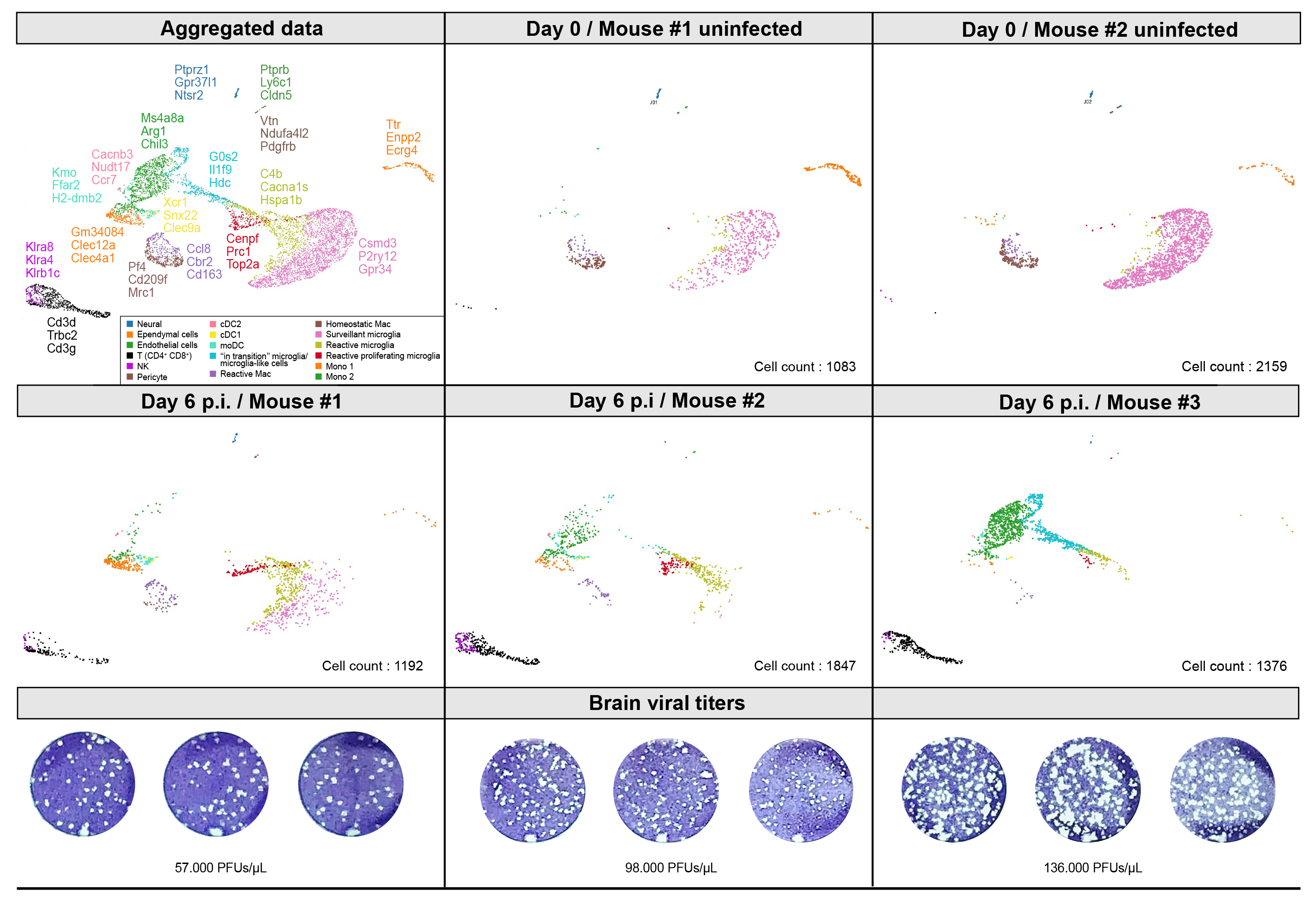

Supplement: Supplementary file 3 — Additional file 3: Figure S3. UMAP visualization of aggregated and individual scRNA-seq data of two uninfected and three infected mice on day 6 p.i., showing different cell sub-clusters for each individual sample. Representative plaque assay wells used to determine the viral titers are provided below UMAP. [file 12974_2022_2437_MOESM3_ESM.tif]

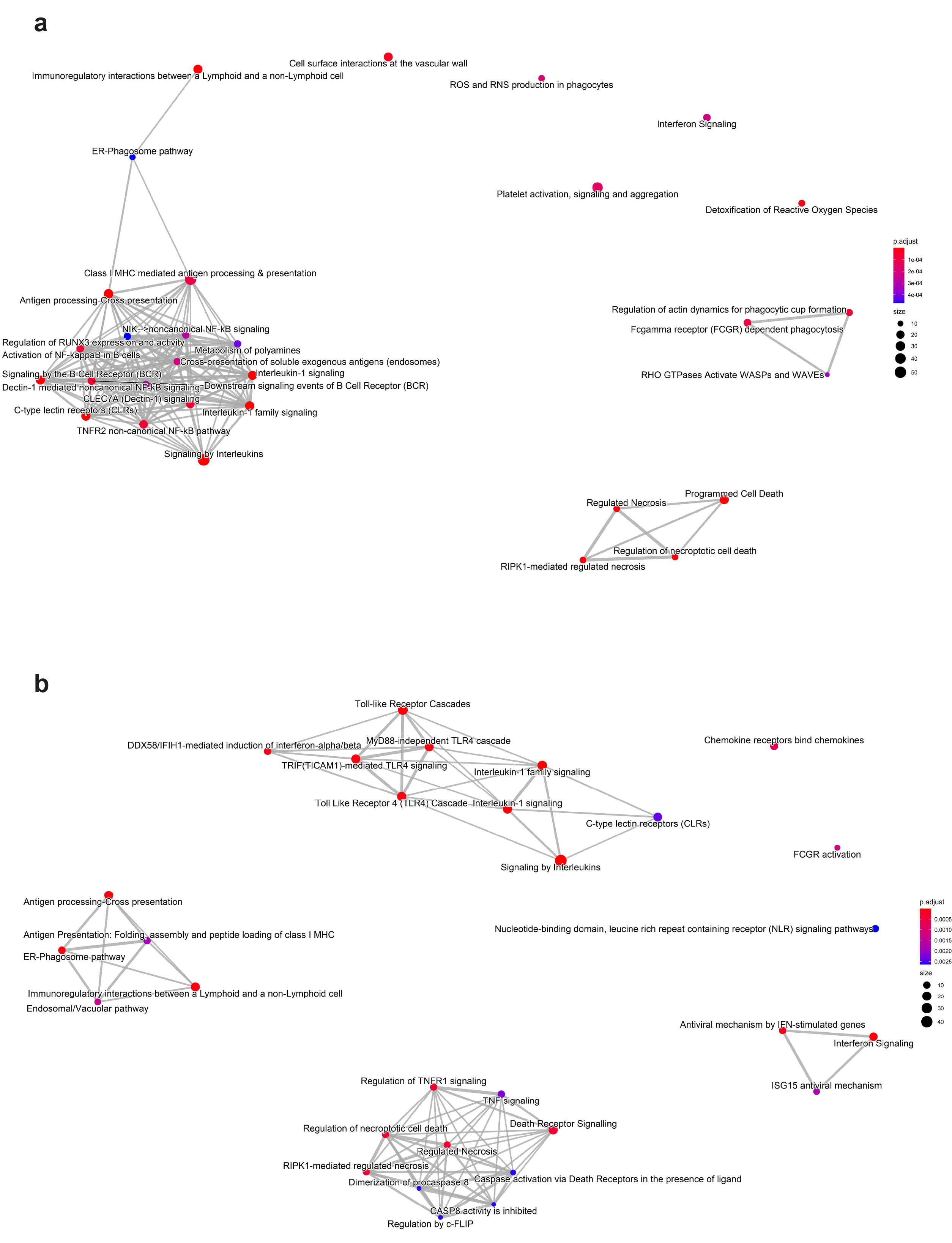

Supplement: Supplementary file 4 — Additional file 4: Figure S4. Enrichment maps of Reactome pathways for Mono2 (a) and “in transition” microglia/microglia-like cells (b). The size of each dot represents the number of genes in each cell sub-cluster, and the color of each dot indicates the normalized enrichment score of each pathway (p-adjust). Pathways that were not significantly enriched (Q value ≥ 0.05, Benjamini–Hochberg correction) are not displayed. [file 12974_2022_2437_MOESM4_ESM.tif]

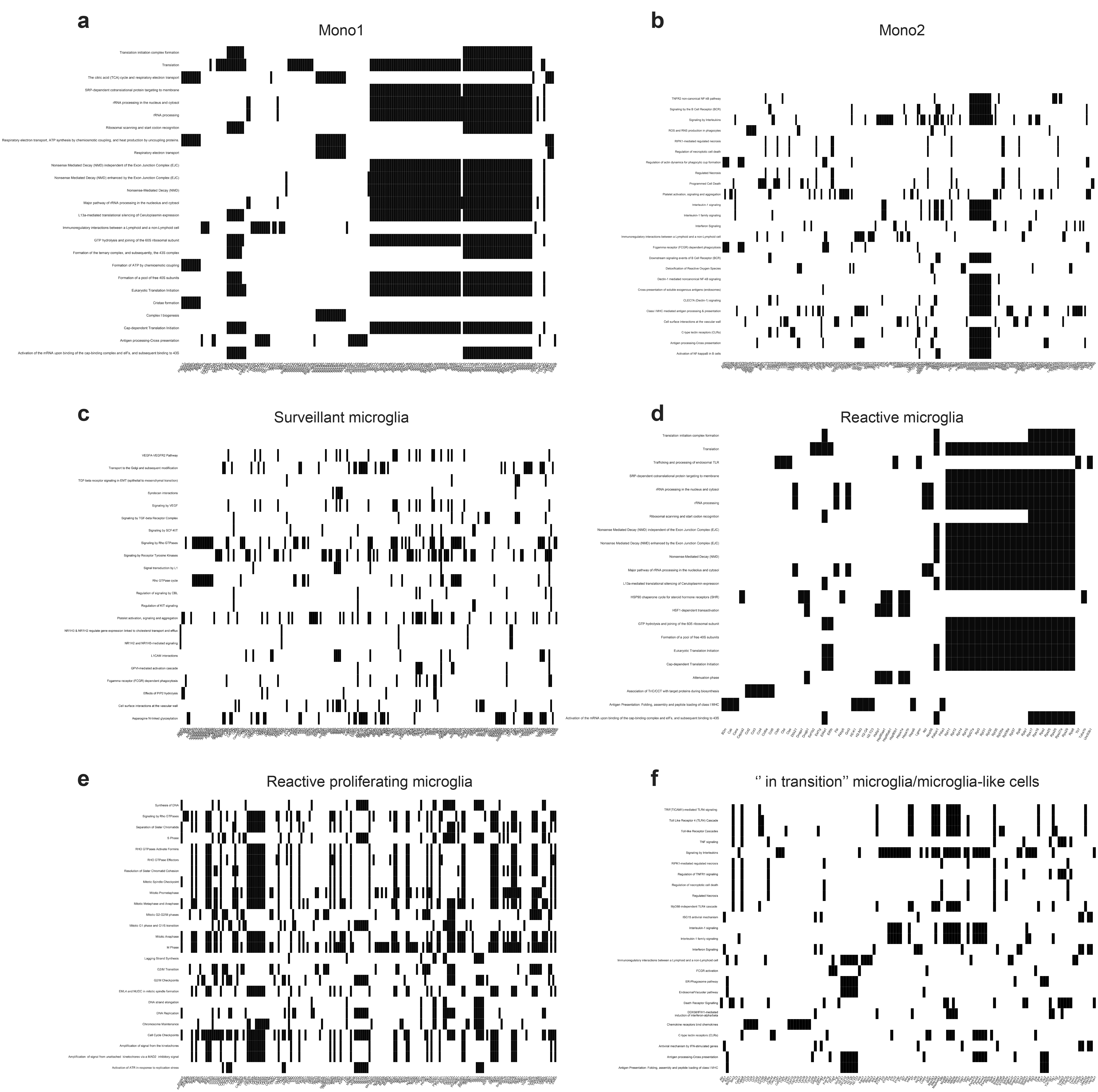

Supplement: Supplementary file 5 — Additional file 5: Figure S5. Heatplots for monocytes and microglial cell sub-clusters. Heatmap-like functional classification of different genes into Reactome pathways were performed for (a) Mono1, (b) Mono2, (c) surveillant, (d) reactive, (e) reactive proliferating, and (f) “in transition” microglia/microglia-like cells. [file 12974_2022_2437_MOESM5_ESM.tif]
